# Artificial intelligence for MRI stroke detection: a systematic review and meta-analysis

**DOI:** 10.1186/s13244-024-01723-7

**Published:** 2024-06-24

**Authors:** Jonas Asgaard Bojsen, Mohammad Talal Elhakim, Ole Graumann, David Gaist, Mads Nielsen, Frederik Severin Gråe Harbo, Christian Hedeager Krag, Malini Vendela Sagar, Christina Kruuse, Mikael Ploug Boesen, Benjamin Schnack Brandt Rasmussen

**Affiliations:** 1 Research and Innovation Unit of Radiology, Odense University Hospital, University of Southern Denmark, Odense, Denmark; 2 Research Unit of Radiology, Aarhus University Hospital, Aarhus University, Aarhus, Denmark; 3 Research Unit for Neurology, Odense University Hospital, University of Southern Denmark, Odense, Denmark; 4 Department of Computer Science, University of Copenhagen, Copenhagen, Denmark; 5 Radiological AI Test Center, Copenhagen University Hospital—Bispebjerg, Frederiksberg, Herlev and Gentofte Hospital, Copenhagen, Denmark; 6 Department of Radiology, Copenhagen University Hospital—Herlev and Gentofte, Copenhagen, Denmark; 7 Institute of Clinical Medicine, University of Copenhagen, Copenhagen, Denmark; 8 Department of Neurology, Copenhagen University Hospital—Herlev and Gentofte, Copenhagen, Denmark; 9 Department of Neurology, Copenhagen University Hospital—Rigshospitalet, Copenhagen, Denmark; 10 Department of Radiology, Copenhagen University Hospital—Bispebjerg and Frederiksberg, Copenhagen, Denmark; 11 Centre for Clinical Artificial Intelligence, Odense University Hospital, University of Southern Denmark, Odense, Denmark

**Keywords:** Artificial intelligence, Magnetic resonance imaging, Stroke, Systematic review, Meta-analysis

## Abstract

**Objectives:**

This systematic review and meta-analysis aimed to assess the stroke detection performance of artificial intelligence (AI) in magnetic resonance imaging (MRI), and additionally to identify reporting insufficiencies.

**Methods:**

PRISMA guidelines were followed. MEDLINE, Embase, Cochrane Central, and IEEE Xplore were searched for studies utilising MRI and AI for stroke detection. The protocol was prospectively registered with PROSPERO (CRD42021289748). Sensitivity, specificity, accuracy, and area under the receiver operating characteristic (ROC) curve were the primary outcomes. Only studies using MRI in adults were included. The intervention was AI for stroke detection with ischaemic and haemorrhagic stroke in separate categories. Any manual labelling was used as a comparator. A modified QUADAS-2 tool was used for bias assessment. The minimum information about clinical artificial intelligence modelling (MI-CLAIM) checklist was used to assess reporting insufficiencies. Meta-analyses were performed for sensitivity, specificity, and hierarchical summary ROC (HSROC) on low risk of bias studies.

**Results:**

Thirty-three studies were eligible for inclusion. Fifteen studies had a low risk of bias. Low-risk studies were better for reporting MI-CLAIM items. Only one study examined a CE-approved AI algorithm. Forest plots revealed detection sensitivity and specificity of 93% and 93% with identical performance in the HSROC analysis and positive and negative likelihood ratios of 12.6 and 0.079.

**Conclusion:**

Current AI technology can detect ischaemic stroke in MRI. There is a need for further validation of haemorrhagic detection. The clinical usability of AI stroke detection in MRI is yet to be investigated.

**Critical relevance statement:**

This first meta-analysis concludes that AI, utilising diffusion-weighted MRI sequences, can accurately aid the detection of ischaemic brain lesions and its clinical utility is ready to be uncovered in clinical trials.

**Key Points:**

- There is a growing interest in AI solutions for detection aid.
- The performance is unknown for MRI stroke assessment.
- AI detection sensitivity and specificity were 93% and 93% for ischaemic lesions.
- There is limited evidence for the detection of patients with haemorrhagic lesions.
- AI can accurately detect patients with ischaemic stroke in MRI.

**Graphical Abstract:**

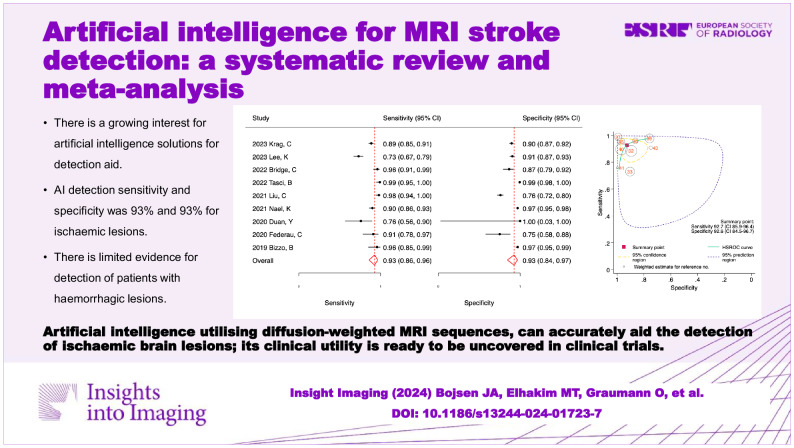

## Introduction

Stroke is an acute onset of focal neurologic symptoms due to of vascular origin from the central nervous system. It is a clinical diagnosis and brain imaging is needed to differentiate between ischaemic and haemorrhagic aetiology. Computed tomography (CT) has for years been the de facto standard imaging modality due to its availability and speed with current guidelines recommending intravenous thrombolysis for ischaemic stroke within 4.5 h of known onset [1, 2]. Presently, many advanced institutions are shifting towards magnetic resonance imaging (MRI) even in the acute diagnosis of stroke. MRI has superior sensitivity and can identify acute ischaemia with unknown stroke onset that is potentially reversible with revascularisation, e.g. by demonstrating a mismatch between diffusion-weighted imaging (DWI) and fluid-attenuated inversion recovery (FLAIR) negative sequences [1–4]. MRI is also highly useful in cases of uncertainty as to a stroke diagnosis. Moreover, MRI optimisation has enabled patient treatment flows similar to those achieved using brain CT regarding, e.g. door-to-needle time [5]. There is increased use of medical imaging including MRI in the healthcare system [6, 7], a trend that is expected to continue in the future [8]. The increasing burden on radiological departments is not predicted to be backed with an equivalent increase in radiologists and it is therefore highly likely that increased MRI use will lead to longer response times or increased error rates [9, 10]. To counterbalance this for stroke diagnosis, artificial intelligence (AI) has been proposed as a technology to enhance the radiology workflow [11–13].

The detection properties of AI can be used in a multitude of workflows including triaging, detection aid, MRI protocol selection, and contrast agent admission decisions. Several studies have reviewed AI for stroke imaging, but these are either applied to CT, are unsystematic, or with a scope too wide to properly elucidate stroke detection in MRI [11–20].

This systematic review aims to assess the performance of AI for automated stroke detection in brain MRI. The objectives of the review are to: (1) estimate the current detection performance for clinically representative studies, (2) characterise the studies, their respective AI algorithms, and whether they have received the European Conformity mark (CE) or received the US Food and Drug Administration (FDA) approval, and (3) utilise the minimum information about clinical artificial intelligence modelling (MI-CLAIM) checklist to characterise reporting trends [21]. For this study, only lesions confirmable in images and compatible with stroke lesions are examined and will onward be mentioned as either ischaemic stroke type or haemorrhagic stroke type depending on their radiological appearance.

## Materials and methods

The review was performed according to the Preferred Reporting of Items for Systematic Reviews and Meta-Analyses (PRISMA) statement [22]. The protocol was prospectively registered with the International Prospective Register of Systematic Reviews (PROSPERO) on 16th November 2021 (CRD42021289748) [23]. Eligibility criteria for inclusion were formed using the participants-intervention-comparator-outcome-study (PICOS) design [24].

### Eligibility criteria

Studies with MRI and AI for stroke assessment, encompassing retrospective, prospective, and diagnostic test studies were included. Participant recruitment strategies were classified as outlined in the Cochrane Handbook [25, 26].

Studies were included if participants were aged 18 years or older, the target condition was stroke or any of its subcategories, and non-stroke patients were used as comparators. At least one of the following had to be reported: (1) sensitivity and specificity, (2) accuracy, or (3) area under the ROC (AUROC) curve.

### Search strategy and information sources

A systematic search was conducted in MEDLINE (Ovid), Embase (Ovid), Cochrane Central, and IEEE Xplore. The search strategy was defined in close cooperation with an information specialist at the local institutional research library. No limitations were made for publication date or language. Subject headings and free text terms relating to the categories MRI, stroke and AI were used. Search blocks were identified for both MRI [27] and stroke [28] through reviews in the Cochrane Library. The reviews from the Cochrane Library were also translated to cover all databases but IEEE Xplore. Due to the restrictions of the IEEE Xplore search machine, the search string was translated to only cover free text terms for this database. Complete search strings for all databases are provided in the online supplementary Table S1. Conference posters and abstracts identified in the search were also eligible. Conference and poster abstracts that were not excluded in the initial screening were followed up by an email enquiry to the corresponding authors for a full record. A reminder e-mail was sent one week after the first if no response was obtained. If no response was obtained after one additional week, they were assessed solely on the information contained in the conference poster or abstract and included based on this if deemed eligible. The systematic searches were updated on 1st November 2023.

### Selection and extraction

**Fig. 1:**
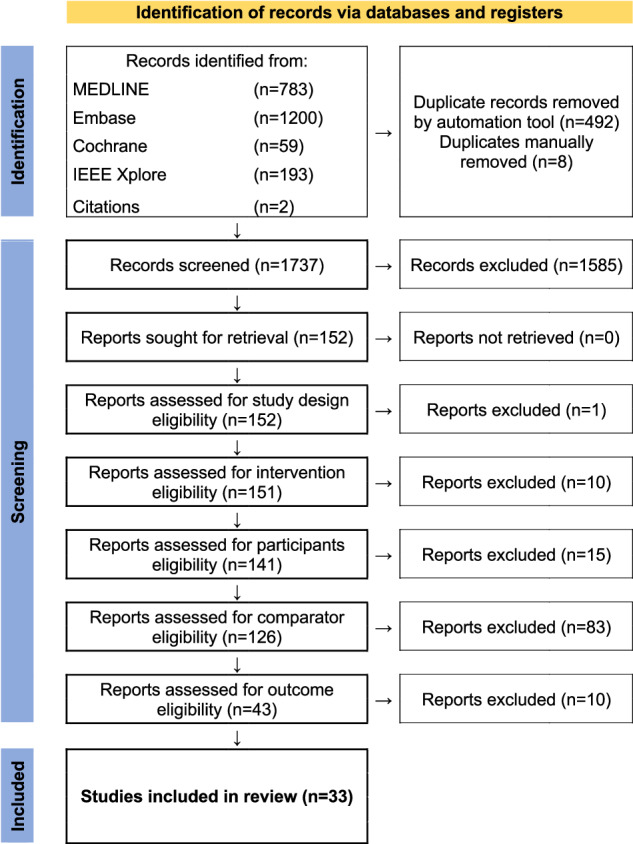
PRISMA chart for the systematic review of AI for MRI stroke detection

All studies were uploaded to EndNote 20 (Clarivate, Philadelphia, PA, USA) and managed with Covidence systematic review software (Veritas Health Innovation, Melbourne, Australia). Duplicates were removed automatically after importation to Covidence. Eligibility was based on the PICOS model as seen in Table 1. Two independent reviewers (J.A.B. and M.T.E.) completed title-abstract and full-text screening and performed bias assessment and data extraction. Any disagreement was resolved through discussion along with arbitration by a third reader (B.S.B.R.). Full-text exclusions were done with reason in categorical order as illustrated in the PRISMA flow chart (Fig. 1). Descriptive data, risk of bias, and results were extracted and handled in consensus between the two primary readers. Risk of bias assessments were performed prior to the assessment of the results to reduce bias in the review. The results collected were sensitivity, specificity, accuracy, and AUROC. Descriptive data collected included Study ID, Study design, Number of participants, Index test, Use of neural network, and FDA approval and CE marking. FDA approval and CE marking status were in addition cross-checked using the Radiology Health AI Register list [29]. Two reviewers (J.A.B. and M.T.E.) independently extracted all data.

**Table 1:** PICOS components for the systematic review of AI for MRI stroke detection

Component	Description
Participants	Patients 18 years of age or older having undergone a brain MRI
Intervention	MRI utilising AI for stroke detection including any stroke subtypes
Comparator	Any manual labelling of stroke or non-stroke MRI diagnosis including any stroke subtypes
Outcome	At least one of the following: -Sensitivity and specificity -Accuracy -AUROC
Study design	Diagnostic test studies utilising either: -A cross sectional design -A case-control design -A cohort design -A randomised trial design

### Risk of bias analysis

For risk of bias analysis, a modified version of the quality assessment for diagnostic accuracy studies 2 (QUADAS-2) tool was used [30]. Modification was done to the index test domain to better accommodate AI. The modified QUADAS-2 tool along with the changes made are illustrated in the online supplementary Table S2.

### Data analysis

Descriptive analysis was done on all included reports. Synthesis of detection results was only performed on reports with an overall low risk of bias. Data on AI performance was abstracted from included studies, or, if not reported, corresponding data were calculated based on available information. A meta-analysis of proportions on true positives (sensitivity) and true negatives (specificity) was performed. A bivariate random effects model with restricted maximum likelihood was used to account for relative heterogeneity. To estimate the general state of AI for detection, a hierarchical summary ROC (HSROC) model was made using the STATA metandi module [31]. The MI-CLAIM checklists [21] were quantitatively synthesised for each study to identify trends in insufficiency in reporting. Trends were analysed overall and for each part, i.e. study design, data and optimisation, model performance, model examination, and reproducibility. Analyses were done using STATA 18 (StataCorp, College Station, TX, USA).

## Results

After duplicate removal, 1738 records were screened by their title and abstract of which 152 were eligible for full-text reading. The total number of included reports was 33 [32–64]. The complete flow of records including reasons for exclusion is illustrated in the PRISMA flow chart (Fig. 1). Full-text reports excluded with reasons for exclusion are listed in Table S3.

### Study characteristics

Twenty-six of the reports were published in 2020 or later. Five reports collected more than one dataset for analysis. Eighteen reports used a case-control design, 12 a cohort design, one collected two datasets of which one set was a cohort and the other a case-control [39], and two reports did not describe their design. No reports used a randomised controlled trial design. Twenty-six studies collected data retrospectively, one study prospectively, and five did not report the method of data collection. One study collected two datasets; one set was retrospective, and no information was provided for the other [37]. For stroke type, 24 reports studied ischaemic stroke, one studied haemorrhagic stroke [63], two had a dataset for both ischaemic and haemorrhagic stroke [40, 49], one studied cerebral venous sinus thrombosis [63], and five reports did not elaborate on stroke type. Four studies performed multicentre data collection [36, 37, 39, 40], but none of them had an external multicentre test set. Descriptive study characteristics for each study are found in Table 2.

**Table 2:** Study characteristics for the systematic review of AI for MRI stroke detection

			Study description			Test sample description				
	Year and first author	Reference	Data collection	Study design	Stroke type	Sample level	Ischaemia	Haemorrhage	Non-stroke	Total
Studies with an overall low risk of bias	2023 Krag, C	[32]	Retrospective	Cohort	Ischaemic	Study	437	Irrelevant	558	995
	2023 Lee, K	[33]	Retrospective	Cohort	Ischaemic	Image	230	Irrelevant	406	636
	2023 Yang, X	[34]	Retrospective	Cohort	CVST	Study* and segment	50 CVST	Irrelevant	50	100
	2023 Wu, Y	[35]	Retrospective	Cohort	Ischaemic	Brain area	Not reported	Irrelevant	Not reported	150
	2022 Bridge, C	[36]	Retrospective	Cohort	Ischaemic	Study	384 168 128* 792	Irrelevant	Set 1: 408 Set 2: 213 Set 3: 119* Set 4: 101	Set 1: 792 Set 2: 381 Set 3: 247* Set 4: 171
	2022 Tasci, B	[37]	Set 1: not reported Set 2: retrospective	Case-control	Ischaemic	Image	Set 1: 77 Set 2: 102*	Irrelevant	Set 1: 37 Set 2: 342*	Set 1: 114 Set 2: 444*
	2022 Qiu, J	[38]	Retrospective	Cohort	Ischaemic	Image	19	Irrelevant	101	120
	2021 Liu, C	[39]	Retrospective	Set 1: cohort Set 2 and 3: case control	Ischaemic	Study	Set 1: 459 Set 2: 140*	Irrelevant	Set 1:499 Set 2: 499*	Set 1: 958 Set 2: 639*
	2021 Nael, K	[40]	Retrospective	Not reported	Both	Study	Set 1: 287* Set 2: 118	Set 1: 78* Set 2: 65	Set 1: 707* Set 2: 867	Set 1: 1072* Set 2: 1050
	2020 Duan, Y	[41]	Retrospective	Cohort	Ischaemic	Study	29	Irrelevant	1	30
	2020 Dørum, E	[42]	Prospective	Case-control	Ischaemic	Study	44	Irrelevant	100	144
	2020 Federau, C	[43]	Retrospective	Case-control	Ischaemic	Study	74	Irrelevant	118	192
	2020 Herzog, L	[44]	Retrospective	Cohort	Ischaemic	Study	Not reported	Irrelevant	102	Not reported
	2019 Bizzo, B	[45]	Retrospective	Cohort	Ischaemic	Study	45	Irrelevant	333	378
	2007 Uchiyama, Y	[46]	Not reported	Cohort	Ischaemic	Image	93	Irrelevant	1053	1156
Studies with an overall high risk of bias	2023 Yaman, S	[47]	Retrospective	Case-control	Ischaemic	Image	102	Irrelevant	342	444
	2022 Arnold, T	[48]	Retrospective	Case-control	Ischaemic	Image	set 1: 143* set 2: 614	Irrelevant	Set 1: 1258* Set 2: 6310	Set 1: 1401* Set 2: 6924
	2022 Eshmawi, A	[49]	Retrospective	Case-control	Both	Image	99	25	54	178
	2022 Guo, Y	[50]	Retrospective	Cohort	Ischaemic	Image	78	Irrelevant	78	156
	2022 Li, J	[51]	Not reported	Case-control	Ischaemic	Study	28	Irrelevant	42	70
	2021 Cetinoglu, Y	[52]	Retrospective	Case-control	Ischaemic	Study	100	Irrelevant	100	200
	2021 Cui, L	[53]	Not reported	Cohort	Ischaemic	Study	Not reported	Irrelevant	Not reported	38
	2021 Hossain, S	[54]	Retrospective	Not reported	Ischaemic	Image	Not reported	Irrelevant	Not reported	25
	2021 Kadry, s	[55]	Retrospective	Case-control	Ischaemic	Image	100	Irrelevant	300	400
	2020 Liu, S	[56]	Not reported	Case-control	Ischaemic	Study	18	Irrelevant	19	37
	2020a Nayak, D	[57]	Retrospective	Case-control	Not reported	Image	Not reported	Not reported	160	200
	2020b Nayak, D	[58]	Retrospective	Case-control	Not reported	Image	Not reported	Not reported	160	200
	2020 Nazari-Farsani, S	[59]	Retrospective	Case-control	Ischaemic	Study	106	Irrelevant	86	192
	2019 Gaidhani, B	[60]	Retrospective	Case-control	Not reported	Study	Not reported	Not reported	51	122
	2019 Nayak, D	[61]	Retrospective	Case-control	Not reported	Image	Not reported	Not reported	160	200
	2019 Ortiz-Ramon, R	[62]	Retrospective	Case-control	Ischaemic	Study	100	Irrelevant	136	236
	2019 Phan, A	[63]	Not reported	Case-control	Haemorrhagic	Image	Irrelevant	42	27	69
	2013 Saritha, M	[64]	Retrospective	Case-control	Not reported	Image	Not reported	Not reported	Not reported	23

*CVT* cerebral venous sinus thrombosis

*Sets used for synthesis of detection performance results

### Setting characteristics

Ten studies had a timeframe setting for stroke onset of 24 h or “acute” with no further specification. Liu et al [39] had longitudinal scan data with patients scanned within both 3 h of symptom onset and again 24 h after symptom onset. None of the other studies utilised a timeframe within 4.5 h, “hyper-acute”, or “FLAIR negative” corresponding to current time or tissue criteria for treatment with thrombolysis. Fourteen studies did not report any definition or specification of the timeframe from onset until the scan. The most used MRI-sequence was FLAIR, T2, T1, and DWI. Two studies utilised functional MRI (fMRI) sequences for assessment [42, 51] and one used time-of-flight [34]. The comparators used in the studies were heterogeneous. Overall, eight studies compared with known normal scans, and three compared with known other pathology. The remaining studies were compared with a mix of patient MRI scans including no pathology, degenerative disorders, and inflammatory disorders. Eighty-five per cent of included studies used a neural network AI with a range of different network architecture backbones. For ten studies, data origin was available in online databases. Of all the studies, only one AI algorithm had received CE marking and none had received FDA approval. The setting characteristics are presented in the online supplementary Table S4.

### Bias assessment

The risk of bias assessment resulted in 15 reports with an overall low risk of bias, out of the 33 included reports. The patient selection domain and the index test domain were responsible for the largest introduction of bias. Seven reports did not describe their reference standard. Although heterogeneous, all studies that reported their reference standard were considered reliable reference standards. Table 3 presents the risk of bias assessment and Table S5 further specifies category bias for each study.

**Table 3:** Risk of bias evaluation for the systematic review of AI for MRI stroke detection

	Reference	Year and first author	Risk of bias			
			/w QUADAS-2			
			Patient selection	Index test	Reference standard	Flow and timing
Overall low risk of bias	[32]	2023 Krag, C	+	+	+	+
	[33]	2023 Lee, K	+	?	+	+
	[34]	2023 Yang, X	+	+	+	+
	[35]	2023 Wu,Y	+	+	+	+
	[36]	2022 Bridge, C	+	+	+	+
	[37]	2022 Tasci, B	?	+	?	+
	[38]	2022 Qiu, J	+	+	+	+
	[39]	2021 Liu, C	+	+	+	+
	[40]	2021 Nael, K	?	+	+	+
	[41]	2020 Duan, Y	+	?	+	+
	[42]	2020 Dørum, E	+	−	+	+
	[43]	2020 Federau, C	?	?	+	+
	[44]	2020 Herzog, L	+	−	+	+
	[45]	2019 Bizzo, B	+	?	+	+
	[46]	2007 Uchiyama, Y	+	+	+	+
Overall high risk of bias	[47]	2023 Yaman, S	−	−	?	+
	[48]	2022 Arnold, T	−	?	+	+
	[49]	2022 Eshmawi, A	−	−	?	+
	[50]	2022 Guo, Y	−	−	?	+
	[51]	2022 Li, J	−	−	+	+
	[52]	2021 Cetinoglu, Y	−	?	+	+
	[53]	2021 Cui, L	?	−	+	+
	[54]	2021 Hossain, S	−	?	?	-
	[55]	2021 Kadry, S	−	?	?	+
	[56]	2020 Liu, S	−	−	?	+
	[57]	2020a Nayak, D	−	?	?	+
	[58]	2020b Nayak, D	−	−	?	+
	[59]	2020 Nazari-Farsani, S	−	?	+	+
	[60]	2019 Gaidhani, B	−	?	?	?
	[61]	2019 Nayak, D	−	?	?	+
	[62]	2019 Ortiz-Ramon, R	−	−	?	+
	[63]	2019 Phan, A	−	?	+	+
	[64]	2013 Saritha, M	−	?	?	+

+: Low risk of bias for category

?: Unclear risk of bias for category

−: High risk of bias for category

### MI-CLAIM assessment

None of the included studies reported to follow the MI-CLAIM checklist, although 17 studies were published in the years after the release of the MI-CLAIM paper from 2020 [21]. Only two studies [32, 34] claimed to follow a reporting standard which was the Standards for Reporting of Diagnostic Accuracy Studies (STARD) guideline [65] and one of those studies [32] additionally followed the Checklist for Artificial Intelligence in Medical Imaging (CLAIM) [66]. The total percentage of reported items was 72%. This was found to be higher in the low risk of bias studies (84% vs 63%). Low-risk and high-risk categories varied significantly in the study design part and in the data and optimisation part with the overall completion rates 100% vs 72% (Chi-squared 13.75; *p* = 0.008) and 93% vs 78% (Chi-squared 7.61; *p* = 0.02), respectively. Only five studies reported all items (except the sharing of code part) [39, 40, 42–44]. The model performance and model examination parts had generally lower rates in reported items with an overall of 64% and 66%, respectively. Five studies, hereof four with a low risk of bias, reported sharing of their code for reproducibility, while the remaining studies did not offer any option to reproduce their results. MI-CLAIM assessment results are presented in the online supplementary Table S6.

### Detection results

The most frequently reported measurements were sensitivity and specificity. Nine of the 33 studies reported AUROC of which four were low risk of bias. Missing values (e.g. accuracy) could be calculated based on other reported values for most studies. Performance ranged from, 51 to 100% for sensitivity, 57 to 100% for specificity, 68 to 99% for accuracy, and 0.83 to 0.98 for AUROC. Liu et al [39] had lower detection rates in the 3-h scans with 96% as compared to 99% in the 24-h scans. Dørum et al [42] utilising fMRI reached random chance detection performance. The single AI examining haemorrhagic stroke from Nael et al [40] performed generally worse than those examining ischaemic stroke. Results for all studies are reported in Table 4. Further notes and clarifications for the results are found in the online supplementary Table S7.

**Table 4:** Performance results for the systematic review of AI for MRI stroke detection

	Year and first author	Reference	*N*	Accuracy	Sensitivity	Specificity	AUROC
Overall low risk of bias	2023 Krag, C	[32]	995	89%	89%	90%	nr
	2023 Lee, K	[33]	636	85%^c^	83%^c^	86%^c^	nr
	2023 Yang, X	[34]	100	92%^b^	96%^b^	88%^b^	0.96^b^
	2023, Wu, Y	[35]	150	93%^c^	90%c	94%^c^	nr
	2022 Bridge, C	[36]	247	92%^c^	96%	87%	0.98
	2022 Tasci, B	[37]	444	99%	99%	99%	nr
	2022 Qiu, J	[38]	120	64%	79%	61%	nr
	2021 Liu, C	[39]	639	81%c	98%	74%	nr
	2021 Nael, K	[40]	1072	95%, 87%^a^	90%, 72%^a^	97%, 88%^a^	0.97, 0.83^a^
	2020 Duan, Y	[41]	30	77%^c^	76%^c^	100%^c^	nr
	2020 Dørum, E	[42]	144	nr	51%	57%	nr
	2020 Federau, C	[43]	192	84%^c^	91%	75%	nr
	2020 Herzog, L	[44]	102	96%	nr	nr	0.89
	2019 Bizzo, B	[45]	378	97%^c^	96%	97%	nr
	2007 Uchiyama, Y	[46]	1056	72%^c^	97%	70%^c^	nr
Overall high risk of bias	2023 Yaman, S	[47]	444	99%	97%	99%	nr
	2022 Arnold, T	[48]	1401	nr	nr	nr	0.94
	2022 Eshmawi, A	[49]	178	99%^c^, 97%^a^	99%^c^, 80%^a^	100%^c^, 100%^a^	nr
	2022 Guo, Y	[50]	156	nr	nr	nr	0.93
	2022 Li, J	[51]	70	80%	61%	93%	0.86
	2021 Cetinoglu, Y	[52]	200	96%	96%	96%	nr
	2021 Cui, L	[53]	38	85%^c^	85%^c^	84%^c^	0.86
	2021 Hossain, S	[54]	25	96%	92%	100%	nr
	2021 Kadry, S	[55]	400	99%	100%	99%	nr
	2020 Liu, S	[56]	37	90%	nr	nr	nr
	2020a Nayak, D	[57]	200	99%	100%	98%	nr
	2020b Nayak, D	[58]	200	97%^c^	88%	99%^c^	nr
	2020 Nazari-Farsani, S	[59]	192	73%	84%	69%	nr
	2019 Gaidhani, B	[60]	122	97%^c^	94%^c^	100%^c^	nr
	2019 Nayak, D	[61]	200	99%	95%	99%	nr
	2019 Ortiz-Ramon, R	[62]	236	68%^c^	72%^c^	65%^c^	0.83
	2019 Phan, A	[63]	69	99%^c^	100%	96%^c^	nr
	2013 Saritha, M	[64]	23	91%	67%	100%	nr

*nr* not reported

^a^Haemorrhagic stroke

^b^Cerebral venous sinus thrombosis

^c^Value calculated from reported outcomes

### Meta-analysis

**Fig. 2:**
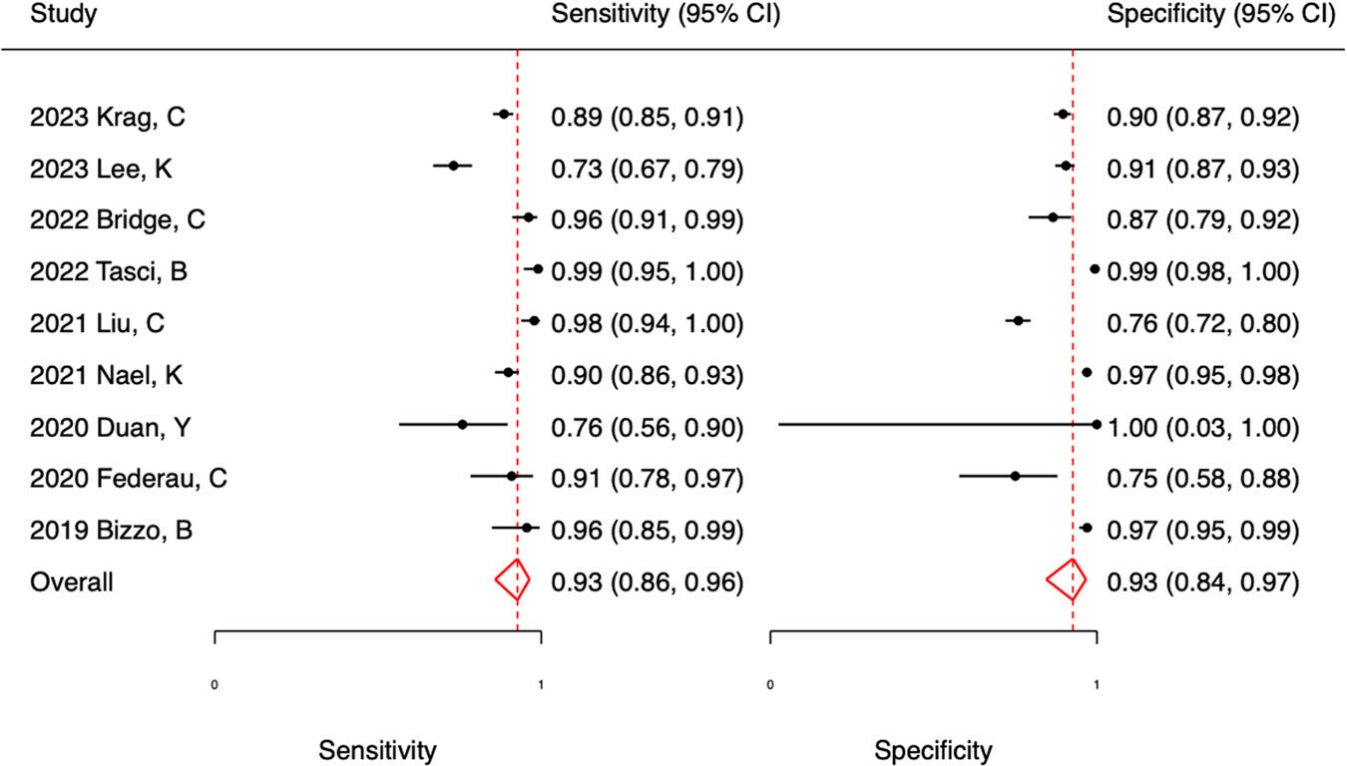
Sensitivity and specificity forest plots for AI in MRI ischaemic stroke detection

**Fig. 3:**
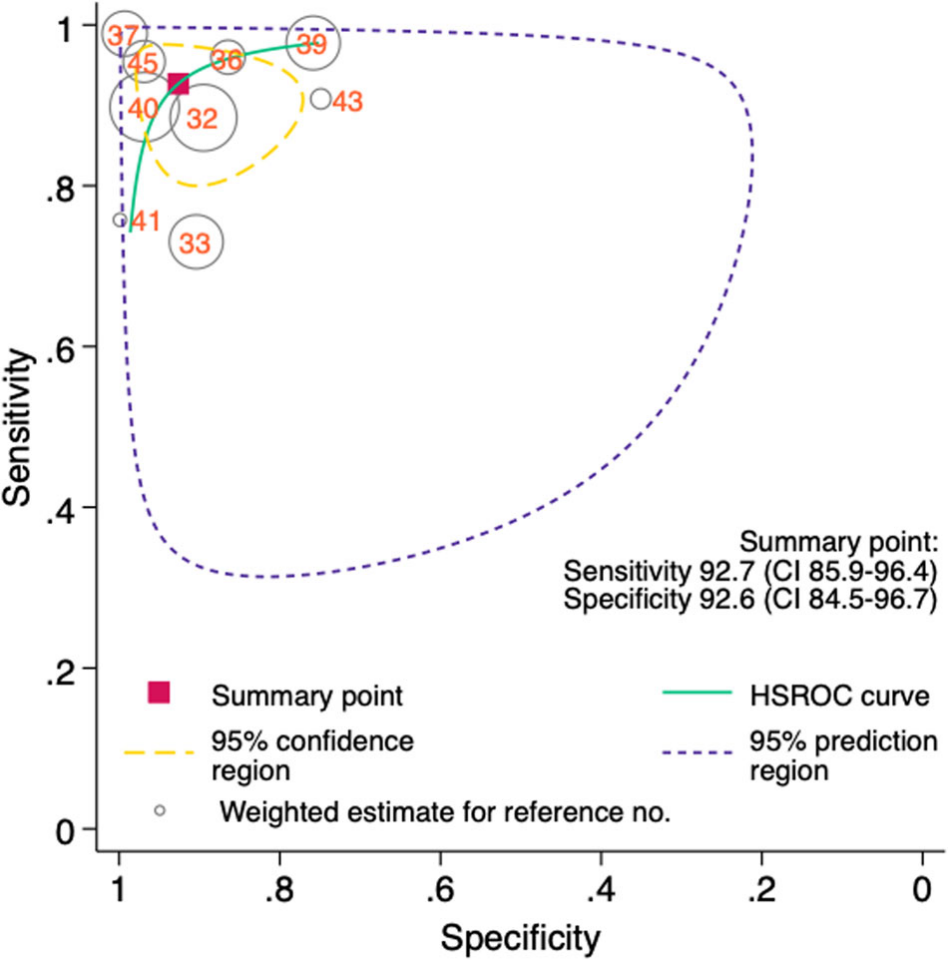
HSROC curve for AI in MRI ischaemic stroke detection

To reduce heterogeneity among the low-risk-bias studies, Yang et al [34], Dørum et al [42], and Uchiyama et al [46] were excluded from the meta-analyses since these studies did not use DWI sequence to detect acute ischaemic stroke lesions. Wu et al [35] were excluded due to insufficient reporting. Forest plot meta-analyses of studies (Fig. 2) revealed an ischaemic stroke detection sensitivity of 93% (CI 86–96%) and specificity of 93% (CI 84–96%). in the HSROC meta-analysis (Fig. 3), the summary point had identical sensitivity and specificity values to corresponding measures in the forest plots. The positive and negative likelihood ratios were 12.6 (CI 5.7–27.7) and 0.079 (CI 0.039–0.159), respectively. The STATA data output from both analyses is presented in Table S8. The literature was not extensive enough to support the conduct of meta-analyses on haemorrhagic stroke.

## Discussion

This systematic review found 33 studies in total assessing AI detection for stroke in MRI. The studies were found to have heterogeneity in the data collection and study design. Most studies examined ischaemic stroke with only a few examining the utility of AI in haemorrhagic stroke. Only one AI algorithm among the included studies had obtained CE marking. The MI-CLAIM assessment revealed insufficiencies in current reporting practice. Based on the nine studies included in the meta-analysis, both ischaemic sensitivity and specificity were 93% with strong likelihood ratios in detecting DWI-positive stroke.

The detection sensitivity in two studies [33, 41] was significantly lower compared to the remaining studies in the meta-analysis. One of them [41] only examined subcortical infarcts which are small vessel based and hence also smaller in lesion volume, which could be the cause, whereas the other [33] separated their stroke scans in single images, which has likely led to some image slices with only a few voxels of actual stroke.

One study [43] achieved significantly lower detection specificity than the remaining studies in the meta-analysis. In this study, the best specificity was obtained by creating synthetic images for training and their algorithm trained without use of the synthetic image was lower at 48%. This could indicate that they had an insufficient amount of available data to train the algorithm to obtain optimum detection performance. The lower detection specificity in another study [39] could likely be due to the set threshold, as they also present a significantly higher detection sensitivity.

One study [37] achieved significantly higher results both in terms of sensitivity and specificity. The most likely reason for this was their utilisation of multiple AI algorithms in a combined iterative majority voting. This practice may be better in terms of raw performance, however costly in terms of computational power requiring much more time to process and large expensive computer setups, which can be difficult to obtain in a clinical setup.

### Reporting of AI studies

Sensitivity, specificity, and accuracy were the most reported outcomes. A measurement of AUROC was not available for most studies. Although FDA-approved and/or CE-marked solutions do exist [29], this systematic review only found one report of such a solution. Therefore, the performance of these commercial AI solutions in clinical practice cannot be extrapolated. The MI-CLAIM checklist was applied to collect the minimum information needed to compare the capabilities of AI and reproduce the results. However, several other relevant reporting guidelines exist, such as the CLAIM guideline, which is a more comprehensive checklist, and the specific AI version of the STARD guideline, which is in the works [65–67]. Given that only five of the 33 reports managed to inform on all MI-CLAIM fields, future studies should follow a relevant checklist for their studies to ensure good reporting practice in research.

### Clinical relevance

Current stroke AI solutions are intended for decision support, as opposed to replacing medical staff [29, 68]. Another task of dismissing the AI false positive scans will be needed, which could prove time-consuming. Additionally, the impact of AI on the decisiveness of radiologists has been investigated in other fields of medical imaging. Mehrizi et al [69] piloted a study for AI support in mammography showing radiologists' evaluations were more prone to be erroneous when the AI made erroneous suggestions.

To assist the assessment before the implementation of an AI solution in a clinical setting, the recently developed model for assessing AI in medical imaging (MAS-AI) could be useful [70]. MAS-AI uses a holistic approach to match different AI algorithms and intended usage scenarios to help support decision-making. How AI affects patient prognosis and the diagnostic work-up routine of stroke patients has not been the scope of this review, but clinical trials examining such are needed prior to implementation.

### Clinical stroke vs radiologically confirmed stroke

Stroke is a clinical diagnosis and occasionally the pathology of interest is invisible on MRI [71]. Furthermore, patients could be suffering from a transient ischaemic attack, where ischaemic lesions often are not visible. Considering the findings in this review, all the included studies utilised image evaluation by one or more medical doctors or the radiology report as their reference standard. It would be of interest to evaluate whether AI possesses the ability to detect strokes not apparent on MRI for the reporting radiologist.

### Limitations

A large proportion of the included studies applied a case-control design, and none were randomised controlled trials. Furthermore, only a small proportion of the studies underwent analysis on external data, which introduces selection bias. We identified only five studies using external datasets for testing [32, 36, 37, 39, 40]. Systematic reviews for AI in other radiological fields have shown that AI performance decreases when tested on externally collected data [72, 73]. Therefore, it is preferable for future AI validation studies to incorporate externally collected, clinically representable datasets and this step is crucial for any AI prior to clinical use.

Limited data was available for evaluating the influence of time from stroke onset to scan on AI detection performance. However, data from the one study available suggests caution must be made for scans with a time of onset below 3 h, as it could negatively affect the AI detection performance.

This systematic review is possibly affected by reporting bias from selected outcome reporting and publication bias. Ideally, the QUADAS-AI tool would have been fitting in this context, but it is still under development [74]. Instead, we used the currently available QUADAS-2, which we modified in an effort to address established shortcomings of this tool in the context of evaluating AI [74, 75]. However, it is possible our modifications reduced the validity of QUADAS-2. Lastly, the topic of this systematic review is under rapid development, as illustrated by the fact that a large proportion of the studies included were published within the last three years. Major developments in the field in the near future are foreseeable which will necessitate updates of this meta-analysis.

## Conclusion

The current AI detection performance of ischaemic stroke in MRI is usable as a diagnostic test. Further investigation is needed to elucidate AI detection of haemorrhagic stroke. Most AI technologies are based on neural networks. There are reporting gaps, mainly in the reporting of AI model performance and examination, and future AI studies should utilise a reporting guideline to improve validity. The clinical usability is yet to be investigated.

### Supplementary information

ELECTRONIC SUPPLEMENTARY MATERIAL

## Data Availability

Datasheets used for meta-analyses are available upon request from the corresponding author.

## Abbreviations

AI: Artificial intelligence
AUROC: Area under the receiver operating characteristics curve
CE: European conformity
CLAIM: Checklist for artificial intelligence in medical imaging
CT: Computed tomography
DWI: Diffusion-weighted imaging
FDA: Food and drug administration
FLAIR: Fluid-attenuated inversion recovery
fMRI: Functional magnetic resonance imaging
HSROC: Hierarchical summary receiver operating characteristics
MAS-AI: Model for assessing artificial intelligence in medical imaging
MI-CLAIM: Minimum information about clinical artificial intelligence modelling
MRI: Magnetic resonance imaging
PICOS: Participants-intervention-comparator-outcome-study
PRISMA: Preferred reporting of items for systematic reviews and meta-Analyses
PROSPERO: International prospective register of systematic reviews
QUADAS-2: Quality assessment for diagnostic accuracy studies 2
ROC: Receiver operating characteristics
STARD: Standards for reporting of diagnostic accuracy studies
