# Supplementary material for: Artificial intelligence for MRI stroke detection: a systematic review and meta-analysis
Source: Insights Imaging. 2024 Jun 24;15:160. doi: 10.1186/s13244-024-01723-7 (PMC11196541; doi:10.1186/s13244-024-01723-7)
Supplement: Supplementary file 1 — ELECTRONIC SUPPLEMENTARY MATERIAL [file 13244_2024_1723_MOESM1_ESM.pdf]

Artificial intelligence for MRI stroke detection: a systematic review and meta-analysis

ELECTRONIC SUPPLEMENTARY MATERIAL

Insights Imaging (2024) Bojsen JA, Elhakim MT, Graumann O, et al.

Table S1. Search strings for all databases searched for the systematic review of artificial intelligence for MRI stroke detection.

Ovid MEDLINE(R) ALL 1946 to October 31, 2023

1	Exp Magnetic resonance Imaging/
2	((magnetic resonance or MR or NMR) adj2 (imag* or tomograph* or scan*)).tw.
3	(MRI or MRIs or NMRI).tw.
4	(diffusion weighted imag* or DWI or T2-weighted imag*).tw.
5	1 or 2 or 3 or 4
6	cerebrovascular disorders/ or exp basal ganglia cerebrovascular disease/ or exp brain ischemia/ or exp carotid artery diseases/ or exp cerebrovascular trauma/ or exp intracranial arterial diseases/ or exp intracranial arteriovenous malformations/ or exp "intracranial embolism and thrombosis"/ or exp intracranial hemorrhages/ or stroke/ or exp brain infarction/ or vasospasm, intracranial/
7	(stroke or poststroke or post-stroke or cerebrovasc$ or brain vasc$ or cerebral vasc$ or cva$ or apoplex$ or SAH).tw.
8	((brain$ or cerebr$ or cerebell$ or intracran$ or intracerebral) adj5 (isch?emi$ or infarct$ or thrombo$ or emboli$ or occlus$)).tw.
9	((brain$ or cerebr$ or cerebell$ or intracerebral or intracranial or subarachnoid) adj5 (haemorrhage$ or hemorrhage$ or haematoma$ or hematoma$ or bleed$)).tw.
10	((transi$ adj3 isch?em$ adj3 attack$) or TIA$1).tw.
11	6 or 7 or 8 or 9 or 10
12	exp Artificial Intelligence/
13	((artificial or machine or deep) adj6 (intelligence or learning)).tw.
14	12 or 13
15	5 and 11 and 14

Embase Classic+Embase 1947 to 2023 October 31

1. Exp Magnetic resonance Imaging/
2. ((magnetic resonance or MR or NMR) adj2 (imag* or tomograph* or scan*)).tw.
3. (MRI or MRIs or NMRI).tw.
4. (diffusion weighted imag* or DWI or T2-weighted imag*).tw.
5. 1 or 2 or 3 or 4
6. cerebrovascular disease/ or exp basal ganglion hemorrhage/ or exp brain hematoma/ or exp brain hemorrhage/ or exp brain infarction/ or exp brain ischemia/ or exp carotid artery disease/ or cerebral artery disease/ or exp cerebrovascular accident/ or exp intracranial aneurysm/ or exp occlusive cerebrovascular disease/ or vertebrobasilar insufficiency/ or stroke/ or stroke patient/ or stroke unit/
7. (stroke or poststroke or post-stroke or cerebrovasc$ or brain vasc$ or cerebral vasc$ or cva$ or apoplex$ or SAH).tw.
8. ((brain$ or cerebr$ or cerebell$ or intracran$ or intracerebral) adj5 (isch?emi$ or infarct$ or thrombo$ or emboli$ or occlus$)).tw.
9. ((brain$ or cerebr$ or cerebell$ or intracerebral or intracranial or subarachnoid) adj5 (haemorrhage$ or hemorrhage$ or haematoma$ or hematoma$ or bleed$)).tw.
10. ((transi$ adj3 isch?em$ adj3 attack$) or TIA$1).tw.
11. 6 or 7 or 8 or 9 or 10
12. exp Artificial Intelligence/
13. ((artificial or machine or deep) adj6 (intelligence or learning)).tw.
14. 12 or 13
15. 5 and 11 and 14

Cochrane Central Register of Controlled Trials Issue 10 of 12, October 2023

#1	MeSH descriptor: [Magnetic Resonance Imaging] explode all trees
#2	((magnetic resonance or MR or NMR) NEAR/2 (imag* or tomograph* or scan*)):ti,ab,kw
#3	(MRI or MRIs or NMRI):ti,ab,kw
#4	(diffusion weighted imag* or DWI or T2-weighted imag*):ti,ab,kw
#5	{OR #1-#4}
#6	MeSH descriptor: [Basal Ganglia Cerebrovascular Disease] this term only
#7	MeSH descriptor: [Brain Ischemia] this term only
#8	MeSH descriptor: [Carotid Artery Diseases] this term only
#9	MeSH descriptor: [Cerebrovascular Trauma] this term only
#10	MeSH descriptor: [Intracranial Arterial Diseases] this term only
#11	MeSH descriptor: [Intracranial Arteriovenous Malformations] this term only
#12	MeSH descriptor: [Intracranial Embolism and Thrombosis] this term only
#13	MeSH descriptor: [Intracranial Hemorrhages] this term only
#14	MeSH descriptor: [Stroke] explode all trees
#15	MeSH descriptor: [Brain Infarction] explode all trees
#16	MeSH descriptor: [Vasospasm, Intracranial] explode all trees
#17	MeSH descriptor: [Cerebrovascular Disorders] explode all trees
#18	(stroke or poststroke or "post-stroke" or cerebrovasc* or brain next vasc* or cerebral next vasc* or cva* or apoplex* or SAH):ti,ab,kw
#19	((brain* or cerebr* or cerebell* or intracran* or intracerebral) near/5 (isch*mi* or infarct* or thrombo* or emboli* or occlus*)):ti,ab,kw
#20	((brain* or cerebr* or cerebell* or intracerebral or intracranial or subarachnoid) near/5 (haemorrhage* or hemorrhage* or haematoma* or hematoma* or bleed*))
#21	{OR #6-#20}
#22	MeSH descriptor: [Artificial Intelligence] explode all trees
#23	((artificial:ti,ab OR machine:ti,ab OR deep:ti,ab) NEAR/6 (intelligence:ti,ab OR learning:ti,ab))
#24	{OR #22-#23}
#25	(#5 AND #21 AND #24)

IEEE Xplore 2004-2023 October 31

(((magnetic resonance OR MR OR NMR OR diffusion weighted OR T2-weighted) NEAR/2 (imag* OR scan*) OR (MRI OR MRIs OR NMRI OR DWI))<br>AND<br>((stroke OR cerebrovascular) OR((brain OR cerebral OR intracranial) NEAR/5 ((ischemic OR infarct OR thrombo* OR emboli* OR occlus*) OR (hemorrhage OR hematoma OR bleed*))))<br>AND<br>((artificial OR machine OR deep) NEAR/6 (intelligence OR learning)))

Table S2. Modified QUADAS-2 for use in the systematic review of artificial intelligence for MRI stroke detection. Parts removed from the original QUADAS-2 are marked with crossed text and parts added are marked with bold text.

Domain 1: Patient selection	
Question 1: Was a consecutive or random sample of patients enrolled?	Yes/no/unclear
Question 2: Was a case-control design avoided?	Yes/no/unclear
Question 3: Did the study avoid inappropriate exclusions?	Yes/no/unclear
Could the selection of patients have introduced bias?	RISK: Low/high/unclear
Domain 2: Index test(s)	
~~Question 1: Were the index test results interpreted without knowledge of the results of the reference standards?~~	~~Yes/no/unclear~~
**Question 1: Is the test set separated from the training set?**	**Yes/no/unclear**
**Question 2: Is the test set externally collected from the training set?**	**Yes/no/unclear**
Question 3: If a threshold was used, was it pre-specified?	Yes/no/unclear
Could the conduct of interpretation of the index test have introduced bias?	RISK: Low/high/unclear
Domain 3: Reference standard	
Question 1: Is the reference standard likely to correctly classify the target condition?	Yes/no/unclear
Question 2: Were the reference standard results interpreted without knowledge of the results from the index test?	Yes/no/unclear
Could the reference standard, its conduct, or its interpretation have introduced bias?	RISK: Low/high/unclear
Domain 4: Flow and timing	
Question 1: Was there an appropriate interval between the index test(s) and the reference standard?	Yes/no/unclear
Question 2: Did all patients receive a reference standard **AND** did all patients receive the same reference standard?	Yes/no/unclear ~~Yes/no/unclear~~
Question 3: Were all patients included in the analysis?	Yes/no/unclear
Could the patients flow have introduced bias?	RISK: Low/high/unclear

Table S3. Full text reports excluded with reasons for exclusion in the systematic review of artificial intelligence for MRI stroke detection.

Excluded due to wrong participants:
1. Behera TK, Khan MA, Bakshi S. Brain MR Image Classification Using Superpixel-Based Deep Transfer Learning. IEEE Journal of Biomedical and Health Informatics. 2022((Behera, Bakshi) Department of Computer Science and Engineering, National Institute of Technology Rourkela, Odisha, India(Khan) Computer Science Department, HITEC University, Taxila, Pakistan):1-11. https://dx.doi.org/10.1109/JBHI.2022.3216270
2. Dubost F, Adams H, Yilmaz P, et al. Weakly supervised object detection with 2D and 3D regression neural networks. Medical image analysis. 2020;65(c8s, 9713490):101767. https://dx.doi.org/10.1016/j.media.2020.101767
3. Ghesu FC, Georgescu B, Mansoor A, et al. Contrastive self-supervised learning from 100 million medical images with optional supervision. Journal of Medical Imaging. 2022;9(6):064503. https://dx.doi.org/10.1117/1.JMI.9.6.064503
4. Huang S, Shen Q, Duong TQ. Artificial neural network prediction of ischemic tissue fate in acute stroke imaging. Journal of cerebral blood flow and metabolism : official journal of the International Society of Cerebral Blood Flow and Metabolism. 2010;30(9):1661-70. https://dx.doi.org/10.1038/jcbfm.2010.56
5. Hussein R, Zhao MY, Shin D, et al., editors. Multi-task Deep Learning for Cerebrovascular Disease Classification and MRI-to-PET Translation2022.
6. Muhammad A, Guojun W. Segmentation of Calcification and Brain Hemorrhage with Midline Detection. 2017 IEEE International Symposium on Parallel and Distributed Processing with Applications and 2017 IEEE International Conference on Ubiquitous Computing and Communications (ISPA/IUCC). 2017:1082-90. 10.1109/ISPA/IUCC.2017.00164
7. Sanchesa P, Meyer C, Vigon V, Naegel B. Cerebrovascular Network Segmentation of MRA Images With Deep Learning. 2019 IEEE 16th International Symposium on Biomedical Imaging (ISBI 2019). 2019:768-71. 10.1109/ISBI.2019.8759569
8. Sathish R, Rajan R, Vupputuri A, Ghosh N, Sheet D. Adversarially Trained Convolutional Neural Networks for Semantic Segmentation of Ischaemic Stroke Lesion using Multisequence Magnetic Resonance Imaging. Annual International Conference of the IEEE Engineering in Medicine and Biology Society IEEE Engineering in Medicine and Biology Society Annual International Conference. 2019;2019(101763872):1010-3. https://dx.doi.org/10.1109/EMBC.2019.8857527
9. Talo M, Yildirim O, Baloglu UB, Aydin G, Acharya UR. Convolutional neural networks for multi-class brain disease detection using MRI images. Computerized medical imaging and graphics : the official journal of the Computerized Medical Imaging Society. 2019;78(cmi, 8806104):101673. https://dx.doi.org/10.1016/j.compmedimag.2019.101673
10. Zhang Y, Hu Q, Guo Z, Xu J, Xiong K. Multi-Class Brain Images Classification Based on Reality-Preserving Fractional Fourier Transform and Adaboost. 2018 IEEE 3rd International Conference on Image, Vision and Computing (ICIVC). 2018:444-7. 10.1109/ICIVC.2018.8492732
11. Assam M, Kanwal H, Farooq U, Shah SK, Mehmood A, Choi GS. An Efficient Classification of MRI Brain Images. IEEE Access. 2021;9:33313-22. 10.1109/ACCESS.2021.3061487
12. Talo M, Baloglu UB, Yildirim O, Rajendra Acharya U. Application of deep transfer learning for automated brain abnormality classification using MR images. Cognitive Systems Research. 2019;54((Talo, Baloglu, Yildirim) Department of Computer Engineering, Munzur University, Tunceli, Turkey(Rajendra Acharya) Department of Electronics and Computer Engineering, Ngee Ann Polytechnic, Singapore(Rajendra Acharya) Department of Biomedical Engineering):176-88. http://dx.doi.org/10.1016/j.cogsys.2018.12.007
13. A V, A S, Metun M, P R, editors. Supervised Machine Learning System Based Segmentation and Classification of Strokes Using Deep Learning Techniques2022.
14. Patil A, Govindaraj S, editors. An AI Enabled Framework for MRI-based Data Analytics for Efficient Brain Stroke Detection2023.
15. Yadav PK, Patel S, Das S, Singh S, Sharma A, editors. MRI Based Automatic Brain Stroke Detection Using CNN Models Improved with Model Scaling2023.

Excluded due to wrong intervention:

1. Ahmed KS, Shariar KS, Naim NH, Alam MGR, editors. Intracranial Hemorrhage Detection using CNN-LSTM Fusion Model2022.
2. Alshammari A, Atiyah N, Alaboodi H, Alshammari R. Identification of stroke using deepnet machine learning algorithm. International Journal of Medical Engineering and Informatics. 2023;15(5):416-29. https://dx.doi.org/10.1504/IJMEI.2023.133083
3. Jung S, Whangbo T. Evaluating a Deep-Learning System for Automatically Calculating the Stroke ASPECT Score. 2018 International Conference on Information and Communication Technology Convergence (ICTC). 2018:564-7. 10.1109/ICTC.2018.8539358
4. Koh TS, Wu XY, Cheong LH, Lim CCT. Assessment of perfusion by dynamic contrast-enhanced imaging using a deconvolution approach based on regression and singular value decomposition. IEEE transactions on medical imaging. 2004;23(12):1532-42. 10.1109/TMI.2004.837355
5. Kothala LP, Guntur SR, editors. An efficient False Negative Reduction System for the Identification of Intracranial Hemorrhage2022.
6. Mazurek MH, Parasuram NR, Peng TJ, et al. Detection of Intracerebral Hemorrhage Using Low-Field, Portable Magnetic Resonance Imaging in Patients With Stroke. Stroke. 2023;54(11):2832-41. https://dx.doi.org/10.1161/STROKEAHA.123.043146
7. Nguyen XB, Lee GS, Kim SH, Yang HJ. Self-Supervised Learning Based on Spatial Awareness for Medical Image Analysis. IEEE Access. 2020;8:162973-81. 10.1109/ACCESS.2020.3021469
8. Roder M, Rosa GH, Papa JP, Guimar DC, x00E, es P, editors. Enhancing Shallow Neural Networks Through Fourier-based Information Fusion for Stroke Classification2021.
9. Okashi OMA, Mohammed FM, Aljaaf AJ. An Ensemble Learning Approach for Automatic Brain Hemorrhage Detection from MRIs. 2019 12th International Conference on Developments in eSystems Engineering (DeSE). 2019:929-32. 10.1109/DeSE.2019.00172
10. Cirio JJ, Ciardi C, Buezas M, et al. Implementation of artificial intelligence in hyperacute arterial reperfusion treatment in a comprehensive stroke center. Neurologia Argentina. 2021((Cirio, Ciardi, Buezas, Caballero, Lopez, Lopez, Chasco, Lammertyn) Unidad de Stroke, Clinica La Sagrada Familia-Instituto Medico ENERI, Buenos Aires, Argentina(Diluca) Servicio de Diagnostico por Imagenes, Clinica La Sagrada Familia-Instituto Medico ENER). http://dx.doi.org/10.1016/j.neuarg.2021.07.003

Excluded due to wrong comparator:

1. Aboudi F, Drissi C, Kraiem T, editors. Efficient U-Net CNN with Data Augmentation for MRI Ischemic Stroke Brain Segmentation2022.
2. Amin J, Sharif M, Raza M, Anjum MA, Bukhari SAC. Convolutional neural network with batch normalization for glioma and stroke lesion detection using MRI. Cognitive Systems Research. 2020;59((Amin) Department of Computer Science, University of Wah, Pakistan(Amin, Sharif, Raza) Department of Computer Science, COMSATS University Islamabad, Wah Campus, Pakistan(Anjum) College of EME, NUST, Pakistan(Bukhari) Division of Computer Science, Mathemat):304-11. http://dx.doi.org/10.1016/j.cogsys.2019.10.002
3. Artzi M, Aizenstein O, Jonas-Kimchi T, Myers V, Hallevi H, Ben Bashat D. FLAIR lesion segmentation: application in patients with brain tumors and acute ischemic stroke. European journal of radiology. 2013;82(9):1512-8. https://dx.doi.org/10.1016/j.ejrad.2013.05.029
4. Babu R, Pushpalatha N, editors. An Effective Application of Deep Learning in the Early Diagnosis of Stroke2023.
5. Bao Q, Mi S, Gang B, Yang W, Chen J, Liao Q. MDAN: Mirror Difference Aware Network for Brain Stroke Lesion Segmentation. IEEE journal of biomedical and health informatics. 2022;26(4):1628-39. https://dx.doi.org/10.1109/JBHI.2021.3113460
6. Boldsen JK, Engedal TS, Pedraza S, et al. Better Diffusion Segmentation in Acute Ischemic Stroke Through Automatic Tree Learning Anomaly Segmentation. Frontiers in neuroinformatics. 2018;12(101477957):21. https://dx.doi.org/10.3389/fninf.2018.00021
7. Carone D, Harston Harston G, De Angeli F, Griffanti L, Jenkinson M, Sheerin F. Automated infarct definition using a volume-dependent machine learning approach. European Stroke Journal. 2019;4(Supplement 1):433. http://dx.doi.org/10.1177/2396987319845581
8. Chen G, Li Q, Shi F, Rekik I, Pan Z. RFDCR: Automated brain lesion segmentation using cascaded random forests with dense conditional random fields. NeuroImage. 2020;211(cpp, 9215515):116620. https://dx.doi.org/10.1016/j.neuroimage.2020.116620
9. Chen G, Ru J, Pan Z, et al. MTANS: Multi-Scale Mean Teacher Combined Adversarial Network with Shape-Aware Embedding for Semi-Supervised Brain Lesion Segmentation. NeuroImage. 2021;244((Chen, Ru, Pan, Lin, Lu, Shi) The First Affiliated Hospital of Wenzhou Medical University, Wenzhou 325000, China(Zhou) The State University of New York at Stony Brook, NY 11794, United States(Rekik) BASIRA Lab, Faculty of Computer and Informatics, Istanbu):118568. http://dx.doi.org/10.1016/j.neuroimage.2021.118568
10. Chen L, Bentley P, Rueckert D. Fully automatic acute ischemic lesion segmentation in DWI using convolutional neural networks. NeuroImage Clinical. 2017;15(101597070):633-43. https://dx.doi.org/10.1016/j.nicl.2017.06.016
11. Chen X, You S, Tezcan KC, Konukoglu E. Unsupervised lesion detection via image restoration with a normative prior. Medical image analysis. 2020;64(c8s, 9713490):101713. https://dx.doi.org/10.1016/j.media.2020.101713
12. Daoudi R, Mouelhi A, Sayadi M. Automatic Ischemic Stroke Lesions Segmentation in Multimodality MRI using Mask Region-based Convolutional Neural Network. 2020 4th International Conference on Advanced Systems and Emergent Technologies (IC_ASET). 2020:362-6. 10.1109/IC_ASET49463.2020.9318265
13. Deepa B, Murugappan M, Sumithra MG, Mahmud M, Al-Rakhami MS. Pattern Descriptors Orientation and MAP Firefly Algorithm based Brain Pathology Classification using Hybridized Machine Learning Algorithm. IEEE Access. 2021:1-. 10.1109/ACCESS.2021.3100549
14. Deepa B, Murugappan M, Sumithra MG, Mahmud M, Al-Rakhami MS. Pattern Descriptors Orientation and MAP Firefly Algorithm Based Brain Pathology Classification Using Hybridized Machine Learning Algorithm. IEEE Access. 2022;10:3848-63. 10.1109/ACCESS.2021.3100549
15. Do L-N, Baek BH, Kim SK, Yang H-J, Park I, Yoon W. Automatic Assessment of ASPECTS Using Diffusion-Weighted Imaging in Acute Ischemic Stroke Using Recurrent Residual Convolutional Neural Network. Diagnostics (Basel, Switzerland). 2020;10(10). https://dx.doi.org/10.3390/diagnostics10100803
16. Giacalone M, Rasti P, Debs N, et al. Local spatio-temporal encoding of raw perfusion MRI for the prediction of final lesion in stroke. Medical image analysis. 2018;50(c8s, 9713490):117-26. https://dx.doi.org/10.1016/j.media.2018.08.008
17. Guerrero R, Qin C, Oktay O, et al. White matter hyperintensity and stroke lesion segmentation and differentiation using convolutional neural networks. NeuroImage Clinical. 2018;17(101597070):918-34. https://dx.doi.org/10.1016/j.nicl.2017.12.022
18. Han L, Liu L, Hao Y, Zhang L. Diagnosis and Treatment Effect of Convolutional Neural Network-Based Magnetic Resonance Image Features on Severe Stroke and Mental State. Contrast media & molecular imaging. 2021;2021(101286760):8947789. https://dx.doi.org/10.1155/2021/8947789

19. Ho KC, Scalzo F, Sarma KV, El-Saden S, Arnold CW. A temporal deep learning approach for MR perfusion parameter estimation in stroke. 2016 23rd International Conference on Pattern Recognition (ICPR). 2016:1315-20. 10.1109/ICPR.2016.7899819
20. Ho KC, Speier W, El-Saden S, Arnold CW. Classifying Acute Ischemic Stroke Onset Time using Deep Imaging Features. AMIA Annual Symposium proceedings AMIA Symposium. 2017;2017(101209213):892-901.
21. Hui H, Zhang X, Li F, Mei X, Guo Y. A Partitioning-Stacking Prediction Fusion Network Based on an Improved Attention U-Net for Stroke Lesion Segmentation. IEEE Access. 2020;8:47419-32. 10.1109/ACCESS.2020.2977946
22. Kabir Y, Dojat M, Scherrer B, Forbes F, Garbay C. Multimodal MRI segmentation of ischemic stroke lesions. Annual International Conference of the IEEE Engineering in Medicine and Biology Society IEEE Engineering in Medicine and Biology Society Annual International Conference. 2007;2007(101763872):1595-8.
23. Kamnitsas K, Ledig C, Newcombe VFJ, et al. Efficient multi-scale 3D CNN with fully connected CRF for accurate brain lesion segmentation. Medical image analysis. 2017;36(c8s, 9713490):61-78. https://dx.doi.org/10.1016/j.media.2016.10.004
24. Karthik R, Menaka R, Hariharan M, Won D. Ischemic Lesion Segmentation using Ensemble of Multi-Scale Region Aligned CNN. Computer methods and programs in biomedicine. 2021;200(doh, 8506513):105831. https://dx.doi.org/10.1016/j.cmpb.2020.105831
25. Kim B, Kwon K, Oh C, Park H. Unsupervised anomaly detection in MR images using multicontrast information. Medical physics. 2021. https://dx.doi.org/10.1002/mp.15269
26. Kim Y-C, Lee J-E, Yu I, et al. Evaluation of Diffusion Lesion Volume Measurements in Acute Ischemic Stroke Using Encoder-Decoder Convolutional Network. Stroke. 2019;50(6):1444-51. https://dx.doi.org/10.1161/STROKEAHA.118.024261
27. Kobold J, Vigneron V, Maaref H, et al. Stroke Thrombus Segmentation on SWAN with Multi-Directional U-Nets. 2019 Ninth International Conference on Image Processing Theory, Tools and Applications (IPTA). 2019:1-6. 10.1109/IPTA.2019.8936074
28. Kumar A, Upadhyay N, Ghosal P, et al. CSNet: A new DeepNet framework for ischemic stroke lesion segmentation. Computer methods and programs in biomedicine. 2020;193(doh, 8506513):105524. https://dx.doi.org/10.1016/j.cmpb.2020.105524
29. Lan W, Ai P, Xu Q. Deep-learning-based MRI in the diagnosis of cerebral infarction and its correlation with the neutrophil to lymphocyte ratio. Annals of palliative medicine. 2021;10(11):11370-81. https://dx.doi.org/10.21037/apm-21-1786
30. Lee AR, Woo I, Lee H, Kim N, Kang D-W, Jung SC. Fully automated segmentation on brain ischemic and white matter hyperintensities lesions using semantic segmentation networks with squeeze-and-excitation blocks in MRI. Informatics in Medicine Unlocked. 2020;21((Lee, Woo, Lee, Kim) Department of Convergence Medicine, Biomedical Engineering Research Center, University of Ulsan, College of Medicine, Asan Medical Center, 88 Olympic-Ro 43-Gil, Songpa-Gu, Seoul 05505, South Korea(Kang) Department of Neurology, Univer):100440. http://dx.doi.org/10.1016/j.imu.2020.100440
31. Lee H, Lee E-J, Ham S, et al. Machine Learning Approach to Identify Stroke Within 4.5 Hours. Stroke. 2020;51(3):860-6. https://dx.doi.org/10.1161/STROKEAHA.119.027611
32. Liu L, Kurgan L, Wu F-X, Wang J. Attention convolutional neural network for accurate segmentation and quantification of lesions in ischemic stroke disease. Medical image analysis. 2020;65(c8s, 9713490):101791. https://dx.doi.org/10.1016/j.media.2020.101791
33. Liu Y, Hancock B, Etherton M, et al. Automated classification of clinical MRI stroke datasets with a recurrent convolutional neural network. Neurology Genetics. 2020;6(SUPPL 1):16-7. http://dx.doi.org/10.1212/NXG.0000000000000422
34. Liu Z, Cao C, Ding S, Liu Z, Han T, Liu S. Towards Clinical Diagnosis: Automated Stroke Lesion Segmentation on Multi-Spectral MR Image Using Convolutional Neural Network. IEEE Access. 2018;6:57006-16. 10.1109/ACCESS.2018.2872939
35. Lucas C, Kemmling A, Mamlouk AM, Heinrich MP. Multi-scale neural network for automatic segmentation of ischemic strokes on acute perfusion images. 2018 IEEE 15th International Symposium on Biomedical Imaging (ISBI 2018). 2018:1118-21. 10.1109/ISBI.2018.8363767
36. Maier O, Handels H. Local problem forests: Classifier training for locally limited sub-problems using spectral clustering. 2015 IEEE 12th International Symposium on Biomedical Imaging (ISBI). 2015:806-9. 10.1109/ISBI.2015.7163994
37. Manikandan S, A G, J JSR, editors. Improved Accuracy in Early Identification of Ischaemic Stroke using K- Nearest Neighbors with Support Vector Machine2023.

38. Mena R, Pelaez E, Loayza F, Macas A, Franco-Maldonado H. An artificial intelligence approach for segmenting and classifying brain lesions caused by stroke. Computer Methods in Biomechanics and Biomedical Engineering: Imaging and Visualization. 2023((Mena, Pelaez, Macas) Electrical & Computer Engineering - FIEC, Escuela Superior Politecnica del Litoral - ESPOL University, Guayaquil, Ecuador(Loayza) Mechanical and Production Science Engineering - FIMCP, Escuela Superior Politecnica del Litoral - ESPOL). https://dx.doi.org/10.1080/21681163.2023.2264410
39. Menze BH, Leemput KV, Lashkari D, et al. A Generative Probabilistic Model and Discriminative Extensions for Brain Lesion Segmentation— With Application to Tumor and Stroke. IEEE transactions on medical imaging. 2016;35(4):933-46. 10.1109/TMI.2015.2502596
40. Mok CW, Chung CS. Fully automatic segmentation of ischemic stroke lesion in 3D MRI images using deep convolutional neural networks with adversarial training. International Journal of Computer Assisted Radiology and Surgery. 2018;13(Supplement 1):S181-S2. http://dx.doi.org/10.1007/s11548-018-1766-y
41. Nah HW, Hyeon K. Lesion patterns recognition using 3 dimensional convolutional neural networks for automated classification of stroke subtypes. International Journal of Stroke. 2018;13(2 Supplement 1):140. http://dx.doi.org/10.1177/1747493018789543
42. Nishi H, Oishi N, Ishii A, et al. Predicting clinical outcomes of acute ischemic stroke due to large vessel occlusion: The approach to utilize high-dimensional neuroimaging data with deep learning. Stroke. 2019;50(Supplement 1). http://dx.doi.org/10.1161/str.50.suppl_1.TP83
43. Noguchi T, Higa D, Asada T, et al. Artificial intelligence using neural network architecture for radiology (AINNAR): classification of MR imaging sequences. Japanese journal of radiology. 2018;36(12):691-7. https://dx.doi.org/10.1007/s11604-018-0779-3
44. Paing MP, Tungjitkusolmun S, Bui TH, Visitsattapongse S, Pintavirooj C. Automated Segmentation of Infarct Lesions in T1-Weighted MRI Scans Using Variational Mode Decomposition and Deep Learning. Sensors (Basel, Switzerland). 2021;21(6). https://dx.doi.org/10.3390/s21061952
45. Pan Y, Zhang H, Yang J, et al. Identification and Diagnosis of Cerebral Stroke through Deep Convolutional Neural Network-Based Multimodal MRI Images. Contrast media & molecular imaging. 2021;2021(101286760):7598613. https://dx.doi.org/10.1155/2021/7598613
46. Pinto A, Amorim J, Hakim A, Alves V, Reyes M, Silva CA. Prediction of Stroke Lesion at 90-Day Follow-Up by Fusing Raw DSC-MRI With Parametric Maps Using Deep Learning. IEEE Access. 2021;9:26260-70. 10.1109/ACCESS.2021.3058297
47. Pinto A, Pereira S, Meier R, et al. Combining unsupervised and supervised learning for predicting the final stroke lesion. Medical image analysis. 2021;69(c8s, 9713490):101888. https://dx.doi.org/10.1016/j.media.2020.101888
48. Polson J, Zhang H, Nael K, et al. A Semi-Supervised Learning Framework to Leverage Proxy Information for Stroke MRI Analysis. Annual International Conference of the IEEE Engineering in Medicine and Biology Society IEEE Engineering in Medicine and Biology Society Annual International Conference. 2021;2021(101763872):2258-61. https://dx.doi.org/10.1109/EMBC46164.2021.9631098
49. Praveen GB, Agrawal A, Sundaram P, Sardesai S. Ischemic stroke lesion segmentation using stacked sparse autoencoder. Computers in biology and medicine. 2018;99(doc, 1250250):38-52. https://dx.doi.org/10.1016/j.compbiomed.2018.05.027
50. Pszczolkowski S, Law ZK, Gallagher RG, et al. Automated segmentation of haematoma and perihaematomal oedema in MRI of acute spontaneous intracerebral haemorrhage. Computers in biology and medicine. 2019;106(doc, 1250250):126-39. https://dx.doi.org/10.1016/j.compbiomed.2019.01.022
51. Qamar S, Jin H, Zheng R, Faizan M. Hybrid loss guided densely connected convolutional neural network for Ischemic Stroke Lesion segmentation. 2019 IEEE 5th International Conference for Convergence in Technology (I2CT). 2019:1-5. 10.1109/I2CT45611.2019.9033802
52. Reddy PB, Kolli S, Bandi R. Brain stroke detection using K-means interfaced with fuzzy C-means for improved accuracy and scanning speed. Turkish Journal of Physiotherapy and Rehabilitation. 2021;32(3):2463-70.
53. Rehme AK, Volz LJ, Feis DL, et al. Identifying Neuroimaging Markers of Motor Disability in Acute Stroke by Machine Learning Techniques. Cerebral cortex (New York, NY : 1991). 2015;25(9):3046-56. https://dx.doi.org/10.1093/cercor/bhu100
54. Ryu WS, Kim DE, Kim DM, Kim BJ, Bae HJ. Diagnostic assessment of deep learning algorithms for classification ischemic stroke subtypes. European Stroke Journal. 2019;4(Supplement 1):310-1. http://dx.doi.org/10.1177/2396987319845581
55. Sahayam S, A A, Jayaraman U. A Novel Modified U-shaped 3-D Capsule Network (MUDCap3) for Stroke Lesion Segmentation from Brain MRI. 2020 IEEE 4th Conference on Information & Communication Technology (CICT). 2020:1-6. 10.1109/CICT51604.2020.9312072

56. Shan W, Wu Z, Wang Y. Automatic diagnosis, classification and subtyping of ischemic stroke based on a deep learning system. International Journal of Stroke. 2021;16(2 SUPPL):24. http://dx.doi.org/10.1177/17474930211041949
57. Shen S, Szameitat AJ, Sterr A. Detection of infarct lesions from single MRI modality using inconsistency between voxel intensity and spatial location--a 3-D automatic approach. IEEE transactions on information technology in biomedicine : a publication of the IEEE Engineering in Medicine and Biology Society. 2008;12(4):532-40. https://dx.doi.org/10.1109/TITB.2007.911310
58. Sudharani K, Sarma TC, Prasad KS. Brain stroke detection using K-Nearest Neighbor and Minimum Mean Distance technique. 2015 International Conference on Control, Instrumentation, Communication and Computational Technologies (ICCICCT). 2015:770-6. 10.1109/ICCICCT.2015.7475383
59. Tolhuisen ML, Hoving JW, Koopman MS, et al. Outcome Prediction Based on Automatically Extracted Infarct Core Image Features in Patients with Acute Ischemic Stroke. Diagnostics (Basel, Switzerland). 2022;12(8). https://dx.doi.org/10.3390/diagnostics12081786
60. Tozlu C, Ozenne B, Cho T-H, et al. Comparison of classification methods for tissue outcome after ischaemic stroke. The European journal of neuroscience. 2019;50(10):3590-8. https://dx.doi.org/10.1111/ejn.14507
61. Wang Y, Katsaggelos AK, Wang X, Parrish TB. A deep symmetry convnet for stroke lesion segmentation. 2016 IEEE International Conference on Image Processing (ICIP). 2016:111-5. 10.1109/ICIP.2016.7532329
62. Winder A, d'Esterre CD, Menon BK, Fiehler J, Forkert ND. Automatic arterial input function selection in CT and MR perfusion datasets using deep convolutional neural networks. Medical physics. 2020;47(9):4199-211. https://dx.doi.org/10.1002/mp.14351
63. Winzeck S, Mocking SJT, Bezerra R, et al. Ensemble of Convolutional Neural Networks Improves Automated Segmentation of Acute Ischemic Lesions Using Multiparametric Diffusion-Weighted MRI. AJNR American journal of neuroradiology. 2019;40(6):938-45. https://dx.doi.org/10.3174/ajnr.A6077
64. Wu W, Lu Y, Mane R, Guan C. Deep Learning for Neuroimaging Segmentation with a Novel Data Augmentation Strategy. 2020 42nd Annual International Conference of the IEEE Engineering in Medicine & Biology Society (EMBC). 2020:1516-9. 10.1109/EMBC44109.2020.9176537
65. Xue Y, Farhat FG, Boukrina O, et al. A multi-path 2.5 dimensional convolutional neural network system for segmenting stroke lesions in brain MRI images. NeuroImage Clinical. 2020;25(101597070):102118. https://dx.doi.org/10.1016/j.nicl.2019.102118
66. Zhang H, Polson JS, Nael K, et al. Intra-domain task-adaptive transfer learning to determine acute ischemic stroke onset time. Computerized medical imaging and graphics : the official journal of the Computerized Medical Imaging Society. 2021;90(cmi, 8806104):101926. https://dx.doi.org/10.1016/j.compmedimag.2021.101926
67. Zhang L, Song R, Wang Y, et al. Ischemic Stroke Lesion Segmentation Using Multi-Plane Information Fusion. IEEE Access. 2020;8:45715-25. 10.1109/ACCESS.2020.2977415
68. Zhang R, Zhao L, Lou W, et al. Automatic Segmentation of Acute Ischemic Stroke From DWI Using 3-D Fully Convolutional DenseNets. IEEE transactions on medical imaging. 2018;37(9):2149-60. https://dx.doi.org/10.1109/TMI.2018.2821244
69. Zhang S, Xu S, Tan L, Wang H, Meng J. Stroke Lesion Detection and Analysis in MRI Images Based on Deep Learning. Journal of Healthcare Engineering. 2021;2021((Zhang, Xu, Tan) College of Information Science and Technology, Qingdao University of Science and Technology, Qingdao 266061, China(Wang) Qingdao Municipal Hospital Qingdao, Qingdao 266071, China(Meng) Qilu Hospital of Shandong University, Qingdao 266035):5524769. http://dx.doi.org/10.1155/2021/5524769
70. Zhang X, Jing S, Gao P, et al. Segmentation of Hyperacute Cerebral Infarcts Based on Sparse Representation of Diffusion Weighted Imaging. Computational and mathematical methods in medicine. 2016;2016(101277751):2581676.
71. Zhao B, Liu Z, Liu G, et al. Deep Learning-Based Acute Ischemic Stroke Lesion Segmentation Method on Multimodal MR Images Using a Few Fully Labeled Subjects. Computational and mathematical methods in medicine. 2021;2021(101277751):3628179. https://dx.doi.org/10.1155/2021/3628179
72. Zhao S, Bagce HF, Spektor V, et al. Deep learning-based covert brain infarct detection from multiple MRI sequences. Neurocomputing. 2023;550((Zhao, Bagce, Spektor, Chou, Gao, Yang, Ma, Schwartz, Zhao) Department of Radiology, Columbia University Irving Medical Center, New York 10032, United States(Morales, Manly, Mayeux, Brickman, Gutierrez) Department of Neurology, Columbia University Irving):126464. https://dx.doi.org/10.1016/j.neucom.2023.126464
73. Zhou Y, Huang W, Dong P, Xia Y, Wang S. D-UNet: A Dimension-Fusion U Shape Network for Chronic Stroke Lesion Segmentation. IEEE/ACM Transactions on Computational Biology and Bioinformatics. 2021;18(3):940-50. 10.1109/TCBB.2019.2939522

74. Zoetmulder R, Gavves E, Caan M, Marquering H. Domain- and task-specific transfer learning for medical segmentation tasks. Computer methods and programs in biomedicine. 2022;214(doh, 8506513):106539. https://dx.doi.org/10.1016/j.cmpb.2021.106539
75. Prakash K N B, Gupta V, Bilello M, Beauchamp NJ, Nowinski WL. Identification, segmentation, and image property study of acute infarcts in diffusion-weighted images by using a probabilistic neural network and adaptive Gaussian mixture model. Academic radiology. 2006;13(12):1474-84.
76. Melingi SB, Vijayalakshmi V. A Hybrid Approach for Sub-Acute Ischemic Stroke Lesion Segmentation Using Random Decision Forest and Gravitational Search Algorithm. Current medical imaging reviews. 2019;15(2):170-83. https://dx.doi.org/10.2174/1573405614666180209150338
77. Gupta A, Vupputuri A, Ghosh N. Delineation of Ischemic Core and Penumbra Volumes from MRI using MSNet Architecture. Annual International Conference of the IEEE Engineering in Medicine and Biology Society IEEE Engineering in Medicine and Biology Society Annual International Conference. 2019;2019(101763872):6730-3. https://dx.doi.org/10.1109/EMBC.2019.8857708
78. Kasischke KA, Burgin WS. Ai-guided detection and volumetric analysis of embolic and watershed infarcts on diffusion-weighted mri enables measurement of the absolute and relative ischemic infarct burden. Stroke. 2021;52(SUPPL 1). http://dx.doi.org/10.1161/str.52.suppl-1.P383
79. Gauriau R, Macruz FBC, Junior OL, et al. A deep learning-based model for detecting abnormalities on brain mr images for triaging: Preliminary results from a multisite experience. Radiology: Artificial Intelligence. 2021;3(4):e200184. http://dx.doi.org/10.1148/ryai.2021200184
80. Muda AF, Saad NM, Waeleh N, Abdullah AR, Fen LY. Integration of Fuzzy C-Means with Correlation Template and Active Contour for Brain Lesion Segmentation in Diffusion-Weighted MRI. 2015 3rd International Conference on Artificial Intelligence, Modelling and Simulation (AIMS). 2015:268-73. 10.1109/AIMS.2015.88
81. Al-Masni MA, Kim WR, Kim EY, Noh Y, Kim DH. 3D Multi-Scale Residual Network Toward Lacunar Infarcts Identification From MR Images With Minimal User Intervention. IEEE Access. 2021;9:11787-97. 10.1109/ACCESS.2021.3051274
82. Mehta S, Grabowski TJ, Trivedi Y, Damasio H. Evaluation of voxel-based morphometry for focal lesion detection in individuals. NeuroImage. 2003;20(3):1438-54.
83. Shen S, Szameitat AJ, Sterr A. An improved lesion detection approach based on similarity measurement between fuzzy intensity segmentation and spatial probability maps. Magnetic resonance imaging. 2010;28(2):245-54. https://dx.doi.org/10.1016/j.mri.2009.06.007

Excluded due to wrong outcome:

1. Chen S, Sedghi Gamechi Z, Dubost F, van Tulder G, de Bruijne M. An end-to-end approach to segmentation in medical images with CNN and posterior-CRF. Medical image analysis. 2022;76(c8s, 9713490):102311. https://dx.doi.org/10.1016/j.media.2021.102311
2. Cho J, Zhang J, Spincemaille P, et al. QQ-NET - using deep learning to solve quantitative susceptibility mapping and quantitative blood oxygen level dependent magnitude (QSM+qBOLD or QQ) based oxygen extraction fraction (OEF) mapping. Magnetic resonance in medicine. 2021. https://dx.doi.org/10.1002/mrm.29057
3. Hussein R, Zhao M, Shin D, Guo J, Zaharchuk G. Multi-task deep learning for classifying cerebrovascular diseases and synthesizing PET from multi-contrast MRI. Journal of Cerebral Blood Flow and Metabolism. 2022;42(1 Supplement):63-4. https://dx.doi.org/10.1177/0271678X221096356
4. Jung W, Yoon J, Ji S, et al. Exploring linearity of deep neural network trained QSM: QSMnet. NeuroImage. 2020;211(cpp, 9215515):116619. https://dx.doi.org/10.1016/j.neuroimage.2020.116619
5. Lange O, Meyer-Baese A, Wismueller A. Exploratory data analysis of dynamic cerebral contrast-enhanced perfusion MRI time-series. Proceedings 2005 IEEE International Joint Conference on Neural Networks, 2005. 2005;4:2406-10 vol. 4. 10.1109/IJCNN.2005.1556279
6. Nazari-Farsani S, Nyman M, Karjalainen T, Bucci M, Isojärvi J, Nummenmaa L, editors. Simplified Automated Segmentation of Acute Ischemic Stroke Lesions from Multimodal MRI: A knowledge-based learning approach2019.
7. Yousaf F, Iqbal S, Fatima N, Kousar T, Shafry Mohd Rahim M. Multi-class disease detection using deep learning and human brain medical imaging. Biomedical Signal Processing and Control. 2023;85((Yousaf, Iqbal, Fatima) Department of Computer Science, Bahauddin Zakariya University, Multan, Pakistan(Iqbal) Department of Information Systems, College of Computer Science and Information Technology, King Faisal University, Al-Ahsa, Saudi Arabia(Kousar):104875. https://dx.doi.org/10.1016/j.bspc.2023.104875
8. Devi CA, Rajagopalan SP. Independent component analysis with genetic algorithm feature selection for ischemic stroke classification. Journal of Pure and Applied Microbiology. 2015;9(Special Edition):215-26.
9. To MNN, Kim HJ, Roh HG, Cho Y-S, Kwak JT. Deep regression neural networks for collateral imaging from dynamic susceptibility contrast-enhanced magnetic resonance perfusion in acute ischemic stroke. International journal of computer assisted radiology and surgery. 2020;15(1):151-62. https://dx.doi.org/10.1007/s11548-019-02060-7
10. Huang W, Yang H, Liu X, et al. A Coarse-to-Fine Deformable Transformation Framework for Unsupervised Multi-Contrast MR Image Registration with Dual Consistency Constraint. IEEE transactions on medical imaging. 2021;40(10):2589-99. 10.1109/TMI.2021.3059282

Excluded due to wrong study design:

1. Mounika S, R. S R, editors. Comprehensive Study on RS_FMRI and EEG Using Deep Learning Approach for Brain Stroke2023.

Table S4. Setting and artificial intelligence characteristics of included studies in the systematic review of artificial intelligence for MRI stroke detection.

	Year and First author	Ref	Setting				Artificial intelligence				
			Comparator	Time frame	Reference standard	Sample origin	FDA/CE approval	Classifier type	Neural Network	Architecture name	Sequences used
Overall low risk of bias	2023 Krag C	(32)	non-ischaemic strokes	4w	neuroradiologist	Denmark	CE approved	CNN	yes	not reported	FLAIR, DWI, SWI/T2*GRE
	2023 Lee, K	(33)	normal	24h	2 radiologists in consensus	Taiwan	none	CNN	yes	modified LeNet	DWI
	2023 Yang, X	(34)	non-strokes	not reported	2 neuroradiologists in consensus	China	none	CNN	yes	MSMT-DL	FLAIR, T1, T2
	2023 Wu, Y	(35)	non-strokes	72h	3 neuroradiologists in consensus	not reported	none	CNN	yes	VGG-16 ResNet-50 CBAM-VGG	DWI
	2022 Bridge, C	(36)	non-strokes with no diffusion restriction	acute ischaemic stroke unspecified	radiologists and neurologists	set 1-3: Massachussetts USA set 4: Brazil	none	CNN	yes	UNet	ADC,DWI B1000
	2022 Tasci, B	(37)	set 1: normal diffusion set 2: white matter hyperintensities, atrophy, normal	acute ischaemic stroke unspecified	1 physician	Turkey	none	CNN	yes	ResNet, DenseNet	set 1: ADC, DWI set 2: T2
	2022 Qiu, J	(38)	intracranial stenosis	not reported	2 radiologists in consensus	China	none	CNN	yes	YOLOv5	TOF-MRA
	2021 Liu, C	(39)	transitory ischaemic attack (MRI non-visible stroke)	set 1: early subacute ischaemic stroke unspecified set 2: two scan time frames: 3h and 24h	1 neuroradiologist	set 1: Maryland USA set 2: STIR database	none	CNN	yes	DAGMNet	ADC, DWI

Table S4 continued.

	Year and First author	Ref	Setting: Comparator	Setting: Time frame	Setting: Reference standard	Setting: Sample origin	Artificial intelligence: FDA/CE approval	Artificial intelligence: Classifier type	Artificial intelligence: Neural Network	Artificial intelligence: Architecture name	Artificial intelligence: Sequences used
**Overall low risk of bias continued**	2021 Nael, K	(40)	Normal, abnormals, mass effect, acute infarction (if detection type is acute haemorrhage) or acute haemorrhage (if detection type is acute infarction)	acute ischaemic stroke unspecified; acute haemorrhagic stroke and extra-axial haemorrhage unspecifed	single radiologist re-evaluation of radiology report (for external test set)	not reported	none	CNN	yes	custom	ADC, FLAIR, Trace (Optional: T1, T2, T2*)
	2020 Duan, Y	(41)	lacunes, white matter hyperintensities, microbleeds	not reported	3 expert radiologists	China	none	CNN	yes	UNet	DWI b1000, FLAIR, T1, T2*
	2020 Dørum, E	(42)	normal	14d	radiology report	Norway	none	Shrinkage LDA	no	-	FLAIR, T1, T2* for fMRI
	2020 Federau, C	(43)	normal	24h	three neuro-radiologists in consensus	Switzerland	none	CNN	yes	UNet	DWI b1000
	2020 Herzog, L	(44)	transitory ischaemic attack (MRI non-visible stroke)	not reported	neurologist with aid from radiology report	Switzerland	none	CNN	yes	custom	DWI
	2019 Bizzo, B	(45)	normal, other unknown non-stroke findings (e.g. neoplasia)	acute ischaemic stroke unspecified	radiologist using radiology report	Massachussetts USA	none	CNN	yes	Ynet	ADC, DWI
	2007 Uchiyama, Y	(46)	non-stroke	not reported	two independent neuro-radiologists	Japan	none	MLP	yes	-	T1, T2

Table S4 continued.

	Year and First author	Setting: Ref	Setting: Comparator	Setting: Time frame	Setting: Reference standard	Setting: Sample origin	Artificial intelligence: FDA/CE approval	Artificial intelligence: Classifier type	Artificial intelligence: Neural Network	Artificial intelligence: Architecture name	Artificial intelligence: Sequences used
Overall high risk of bias	2023 Yaman, S	(47)	normal, atrophy, WMI	not reported	specialist radiologist	Turkey	none	CNN	yes	AlexNet GoogleNet DenseNet VGG MobileNet ResNet SqueezeNet	T2
	2022 Arnold, T	(48)	high grade glioma, low grade glioma, multiple sclerosis	subacute ischaemic stroke unspecified	3 neuro-radiologists	ISLES 2015, BRATS 2019, MICCAI 2008, MS-SEG-2016, OASIS3 databases	none	CNN	yes	DenseNet	Axial FLAIR
	2022 Eshmawi, A	(49)	normal	subacute ischaemic stroke unspecified haemorrhagic stroke not reported	not reported	AANLIB	none	CNN	yes	MobileNet CapsuleNet EfficientNet	T2
	2022 Guo, Y	(50)	non-strokes	24h	RAPID	China	none	LDA	no	-	DSC-PWI
	2022 Li, J	(51)	normal	7d	1 radiologist & 1 neurologist	China	none	SVM	no	-	FMRI (slow-4 and slow-5)
	2021 Cetinoglu, Y	(52)	non-ischaemic stroke patients	acute ischaemic stroke unspecified	2 neuro-radiologists	Turkey	none	CNN	yes	MobileNet	dwi
	2021 Cui, L	(53)	non-acute ischaemic stroke patients	24h	clinicians	China	none	CNN	yes	MedicalNet, DeepSym	ADC, DWI, PWI, T1
	2021 Hossain, S	(54)	non-stroke	not reported	not reported	Kaggle.com Radiopedia.org medscape.com	none	LRC	no	-	not reported

Table S4 continued.

	Year and First author	Ref	Setting: Comparator	Setting: Time frame	Setting: Reference standard	Setting: Sample origin	Artificial intelligence: FDA/CE approval	Artificial intelligence: Classifier type	Artificial intelligence: Neural Network	Artificial intelligence: Architecture name	Artificial intelligence: Sequences used
Overall high risk of bias continued	2023 Yaman, S	(55)	normal, glioma	not reported	not reported	ISLES2015, BRATS2015 databases	none	CNN	yes	VGG	FLAIR
	2020 Liu, S	(56)	normal	acute ischaemic stroke unspecified	clinical specialists	China	none	KNN	yes	-	not reported
	2020a Nayak, D	(57)	normal, degenerative, infectious, tumor	not reported	not reported	med.harvard.edu/ AANLIB/	none	RVFL-AE	yes	custom	T2
	2020b Nayak, D	(58)	normal, degenerative, infectious, tumor	not reported	not reported	med.harvard.edu/ AANLIB/	none	K-ELM	yes	custom	T2
	2020 Nazari-Farsani, S	(59)	normal	acute ischaemic stroke unspecified	1 neuroradiologist	Finland	none	SVM	no	-	ADC, DWI, T1
	2019 Gaidhani, B	(60)	normal	not reported	not reported	ATLAS database	none	CNN	yes	LeNet	T1
	2019 Nayak, D	(61)	normal, degenerative, infectious, tumor	not reported	not reported	med.harvard.edu/ AANLIB/	none	Kernel RVFL	yes		T2
	2019 Ortiz-Ramon, R	(62)	age cohort with no acute strokes	2w	not reported	United Kingdom	none	SVM	no	-	FLAIR, T1, T2
	2019 Phan, A	(63)	epidural haematoma, subdural haematoma, subarachnoid haemorrhage	not reported	unspecified number ofspecialists and doctors	Vietnam	none	CNN	yes	custom	not reported
	2013 Saritha, M	(64)	normal, degenerative, infectious, tumor	not reported	not reported	med.harvard.edu/ AANLIB/	none	PNN	yes	custom	T2

CNN: Convolutional neural network, LDA: Linear discriminant analysis, MLP: Multilayer perceptron, SVM: Support vector machine, LRC: Logistic regression classifier, KNN: K-nearest neighbor, RVFL: Random vector functional link, AE: Autoencoder, PNN: Probabilistic neural network, ADC: Apparent diffusion coefficient, DWI: Diffusion weighted images, FLAIR: Fluid attenuated inversion recovery, fMRI: Functional magnetic resonance imaging

Table S5. In-depth risk of bias of included studies in the systematic review of artificial intelligence for MRI stroke detection.

	Year and first author	Patient selection domain	Index test domain	Reference standard domain	Flow and timing domain
Overall low risk of bias	2023 Krag, C	yes<br>yes<br>yes	yes<br>yes<br>yes	yes<br>yes	yes<br>yes<br>yes
	2023 Lee, K	yes<br>yes<br>yes	yes<br>no<br>no	yes<br>yes	yes<br>yes<br>yes
	2023 Yang, X	yes<br>yes<br>yes	yes<br>no<br>yes	yes<br>yes	yes<br>yes<br>yes
	2023 Wu, Y	yes<br>yes<br>yes	yes<br>no<br>unclear	yes<br>yes	yes<br>yes<br>yes
	2022 Bridge, C	yes<br>yes<br>no	yes<br>yes[1]<br>yes	yes<br>yes	yes<br>yes<br>yes
	2022 Tasci, B	unclear<br>unclear<br>yes	yes[2]<br>yes[2]<br>unclear	unclear[3]<br>yes	yes<br>yes<br>yes
	2022 Qiu, J	yes<br>yes<br>yes	yes<br>no<br>yes	yes<br>yes	yes<br>yes<br>yes
	2021 Liu[4]	yes[3]<br>yes[3]<br>unclear	yes[3]<br>yes[3]<br>yes	yes[3]<br>yes	yes<br>yes<br>yes
	2021 Nael, K	unclear<br>unclear<br>yes	yes<br>yes<br>no	yes<br>yes	yes<br>yes<br>yes
	2020 Duan, Y	unclear<br>yes<br>yes	yes<br>no<br>no	yes<br>yes	yes<br>yes<br>yes
	2020 Dørum, E	yes<br>no<br>yes	no<br>no<br>no	yes<br>yes	yes<br>yes<br>yes
	2020 Federau, C	unclear<br>no<br>yes	yes<br>no<br>no	yes<br>yes	yes<br>yes<br>yes
	2020 Herzog, L	unclear<br>yes<br>yes	unclear<br>no<br>no	yes<br>yes	yes<br>yes<br>yes
	2019 Bizzo, B	yes<br>yes<br>yes	unclear<br>unclear<br>no	yes<br>yes	yes<br>yes<br>yes
	2007 Uchiyama, Y	yes<br>yes<br>yes	yes<br>no<br>yes	yes<br>yes	yes<br>yes<br>yes

Table S5 continued.

**Overall high risk of bias**	2023 Yaman, S	no<br>no<br>unclear	no<br>no<br>no	unclear<br>yes	yes<br>yes<br>yes
	2022 Arnold	no<br>no<br>unclear	yes<br>no<br>no	yes<br>unclear	yes<br>yes<br>no
	2022 Eshmawi, A	no<br>no<br>unclear	no<br>no<br>no	unclear<br>unclear	yes<br>unclear<br>yes
	2022 Guo, Y	no<br>yes<br>yes	no<br>no<br>no	unclear<br>yes	yes<br>yes<br>yes
	2022 Li	no<br>no<br>yes	no<br>no<br>unclear	yes<br>yes	yes<br>yes<br>yes
	2021 Cetinoglu	no<br>no<br>yes	yes<br>no<br>unclear	yes<br>yes	yes<br>yes<br>yes
	2021 Cui[5]	unclear<br>yes<br>unclear	no<br>no<br>unclear	yes<br>yes	yes<br>yes<br>yes
	2021 Hossain, S	no<br>no<br>unclear	yes<br>unclear<br>no	unclear<br>unclear	yes<br>no<br>unclear
	2021 Kadry, s	no<br>no<br>unclear	yes<br>no<br>no	unclear<br>unclear	yes<br>unclear<br>yes
	2020 Liu, S	unclear<br>no<br>unclear	no<br>no<br>no	unclear<br>unclear	yes<br>yes<br>yes
	2020a Nayak, D	no<br>no<br>unclear	yes<br>unclear<br>no	unclear<br>unclear	yes<br>unclear<br>yes
	2020b Nayak, D	no<br>no<br>unclear	unclear<br>unclear<br>no	unclear<br>unclear	yes<br>unclear<br>yes
	2020 NazariFarsani, S	no<br>no<br>yes	yes<br>no<br>no	yes<br>yes	yes<br>yes<br>yes
	2019 Gaidhani, B	unclear<br>no<br>unclear	yes<br>no<br>no	unclear<br>no	yes<br>unclear<br>unclear
	2019 Nayak, D	no<br>no<br>unclear	unclear<br>unclear<br>no	unclear<br>unclear	yes<br>yes<br>yes
	2019 Ortiz-Ramon, R	no<br>no<br>unclear	unclear<br>no<br>no	unclear<br>unclear	yes<br>unclear<br>yes
	2019 Phan, A	unclear<br>no<br>unclear	yes<br>no<br>no	yes<br>yes	yes<br>yes<br>yes
	2013 Saritha, M	no<br>no<br>unclear	yes<br>no<br>no	unclear<br>unclear	yes<br>yes<br>yes

[1]for stroke code and international datasets
[2]only dataset 2
[3]no info dataset 2, dataset 1: yes
[4]STIR dataset is a mix of datasets. Insufficient reporting of ground truth labellers, but the STIR steering committee consists of numerous researchers from wellestablished universities.
[5]Ambiguous separation of training and validation. Bias evaluation is of all patients.

Table S6. MI-CLAIM reported items for included studies in the systematic review of artificial intelligence for MRI stroke detection.

	Year and first author	Ref	Study design (Part 1)	Data and optimization (Parts 2, 3)	Model Performance (part 4)	Model Examination (part 5)	Reproducibility (Part 6)	Overall % completed
Studies with an overall low risk of bias	2023 Krag, C	(32)	5/5	4/4	2/3	3/4	No code sharing	82
	2023 Lee, K	(33)	5/5	4/4	1/3	4/4	no code sharing	82
	2023 Yang, X	(34)	5/5	3/4	2/3	4/4	full sharing of code	88
	2023 Wu, Y	(35)	5/5	4/4	1/3	4/5	no code sharing	78
	2022 Bridge, C	(36)	5/5	4/4	3/3	4/5	no code sharing	89
	2022 Tasci, B	(37)	5/5	4/4	2/3	3/5	no code sharing	78
	2022 Qiu, J	(38)	5/5	4/4	2/3	4/4	no code sharing	88
	2021 Liu, C	(39)	5/5	4/4	3/3	4/4	full sharing of code	100
	2021 Nael, K	(40)	5/5	4/4	2/3	4/4	full sharing of code	94
	2020 Duan, Y	(41)	5/5	3/4	2/3	3/4	no code sharing	76
	2020 Dørum, E	(42)	5/5	4/4	3/3	5/5	no code sharing	94
	2020 Federau, C	(43)	5/5	4/4	3/3	4/4	no code sharing	94
	2020 Herzog, L	(44)	5/5	4/4	3/3	4/4	full sharing of code	100
	2019 Bizzo, B	(45)	5/5	3/4	0/3	0/5	no code sharing	44
	2007 Uchiyama, Y	(46)	5/5	3/4	2/3	2/4	no code sharing	71
	Category %		100	93	69	79	29	84
Studies with an overall high risk of bias	2023 Yaman, S	(47)	5/5	3/4	1/3	2/5	no code sharing	61
	2022 Arnold, T	(48)	5/5	4/4	1/3	4/5	no code sharing	78
	2022 Eshmawi, A	(49)	1/5	3/4	1/3	2/5	no code sharing	39
	2022 Guo, Y	(50)	5/5	3/4	1/3	1/5	no code sharing	56
	2022 Li, J	(51)	5/5	2/4	3/3	4/4	full sharing of code	88
	2021 Cetinoglu, Y	(52)	5/5	3/4	3/3	4/4	no code sharing	88
	2021 Cui, L	(53)	5/5	3/4	2/3	4/4	no code sharing	82
	2021 Hossain, S	(54)	3/5	2/4	2/3	1/5	no code sharing	44
	2021 Kadry, s	(55)	4/5	4/4	2/3	2/5	no code sharing	67
	2020 Liu, S	(56)	4/5	3/4	2/3	0/5	no code sharing	50
	2020a Nayak, D	(57)	2/5	3/4	1/3	4/4	no code sharing	59
	2020b Nayak, D	(58)	2/5	2/4	1/3	4/4	no code sharing	53
	2020 Nazari-Farsani, S	(59)	5/5	4/4	3/3	4/4	no code sharing	94
	2019 Gaidhani, B	(60)	3/5	4/4	2/3	2/5	no code sharing	61
	2019 Nayak, D	(61)	2/5	3/4	2/3	2/5	no code sharing	50
	2019 Ortiz-Ramon, R	(62)	4/5	4/4	3/3	4/4	no code sharing	88
	2019 Phan, A	(63)	3/5	3/4	0/3	1/5	no code sharing	39
	2013 Saritha, M	(64)	2/5	3/4	1/3	1/5	no code sharing	39
	Category %		72	78	57	55	6	63
	**Total %**		**85**	**85**	**63**	**66**	**15**	**72**

Table S7. Notes supplementing the results reported in Table 4.

	Year and first author	Ref	Notes
Overall low risk of bias	2023 Krag, C	(32)	Reported for total included patients. Also reported for non-enriched cohort (89;86;90; nr)
	2023 Lee, K	(33)	Calculated using confusion matrix. ACI and PCI strokes were split.
	2023 Yang, X	(34)	MSMT-DL reported (best). Models using other sequences were also reported.
	2023 Wu, Y	(35)	Calculated averages from each reported area using VGG with CBAM for the four small areas.
	2022 Bridge, C	(36)	Results for non-training stroke code test set (largest of externally collected). Also reported primary set (nr; 98; 98; 0,998) international (nr; 100; 98; 0.998) and training hospital set (nr; 89; 95; 0.964)
	2022 Tasci, B	(37)	Results for Dataset 2 and Iterative Majority voting (External and best). Multiple other results are reported.
	2022 Qiu, J	(38)	Calculated using confusion matrix. Occlusion of intracranial artery counts as stroke vs stenosis of any grade.
	2021 Liu, C	(39)	All results for DAGMnet-CH3 (proposed AI). Multiple other AI tested. Results for STIR-2 dataset (sensitivity) and "Testing - not visible" dataset (specificity). Also reported STIR-1 sensitivity (90%) and Test sensitivity (99%)
	2021 Nael, K	(40)	External testing dataset reported. Also reported for internal testing dataset (88; 92; 88; 0.95) and (81*; 89*; 81*; 0.90*)
	2020 Duan, Y	(41)	
	2020 Dørum, E	(42)	LOAD2 used (best). Also reported REST (nr; 47; 56;nr) and LOAD1 (nr; 50; 47; nr)
	2020 Federau, C	(43)	CS40DB used (best). Also reported CDB (nr; 85; 48;nr), S2DB (nr; 77; 68;nr), CS2DB (nr; 80; 76; nr)
	2020 Herzog, L	(44)	FC-NN 3Ch-MC is used (best). Also reported 1D-CNN 3Ch-MC (95; nr; nr; 0.86)
	2019 Bizzo, B	(45)	
	2007 Uchiyama, Y	(46)	Modular classifier is used (best). Also reported single classifier (35; 97; 29; nr).

Table S7 continued.

Overall high risk of bias	2023 Yaman, S	(47)	
	2022 Arnold, T	(48)	F1-score of 76% reported instead. Results for 64mT (Best AUC). Also reported 3T (F1:77; nr; nr; 0.94)
	2022 Eshmawi, A	(49)	Ischemia calculated using confusion matrix. Hemorrhage were contained in the reported total results. Epoc-2000 used (best)
	2022 Guo, Y	(50)	Reported for DA in DRF group_D, where Lasso + PCA_Lasso ties with Lasso + Tsne_Lasso
	2022 Li, J	(51)	Results for combined Slow-4+Slow-5 (Best AUC, accuracy and specificity). Also reported ipsilesional Slow-4 (73; 68; 76; 0.80) (Best sensitivity) and multiple other with lower parameters.
	2021 Cetinoglu, Y	(52)	Results for MobileNetV2 (best). Also reported EfficientNetB0 (93; 93; 93; nr)
	2021 Cui, L	(53)	Results for DeepSym-3D-CNN (best). Also reported for ADC images only (73; 71; 76; 0.701) and DWI+ADC images only (81; 85; 77; 0.843)
	2021 Hossain, S	(54)	
	2021 Kadry, S	(55)	SVM-Cubic VGG16 reported (best). No stroke specific data available for VGG19 or ResNet50. Also reported SoftMax VGG16 (97,5;100;96,67; nr) and SVM-RBF VGG16 (98,25; 100; 97,67; nr)
	2020 Liu, S	(56)	KNN used (best). Also reported BPNN (89,09; nr; nr), PLSR (88,18; nr; nr; nr), EB (86,36; nr; nr; nr)
	2020a Nayak, D	(57)	MD-2 is used (largest dataset). Figure 5 actual and predicted labels have been swapped (denominator must be reference standard). Also reported for MD-1 (100;100;100; nr)
	2020b Nayak, D	(58)	MD-2 FCEntF-I is used (largest dataset with best specificity). Also reported MD-2 FCEntF-II (97; 93; 98; nr), MD-1 FCEntF-I (99; 93; 100; nr) and MD-1 FCEntF-II (97; 87; 100; nr)
	2020 Nazari-Farsani, S	(59)	
	2019 Gaidhani, B	(60)	Discrepancy between reported and calculated accuracy measurement. Also discrepancy for test population size (122 in table vs 126 reported)
	2019 Nayak, D	(61)	MD-2 is used (largest dataset). Also reported MD-1 (97; 93; 98; nr)
	2019 Ortiz-Ramon, R	(62)	Figure readout: TP=54%, TN=70%, FP= 38%, FN=21%. Multiple classifiers are intermixed. For AUROC Multiple parameters are tested. The best is found in table 6 with parameters: SVM, LBP T2W - SS, MIC
	2019 Phan, A	(63)	
	2013 Saritha, M	(64)	

Table S8a. STATA data from the metadta analysis on artificial intelligence for MRI stroke detection.

```
metadta tp fp fn tn, studyid(id)

*********************************** Fitted model ***************************************
    tp ~ binomial(se, tp + fn)
    tn ~ binomial(sp, tn + fp)
    logit(se) = mu_lse + id_lse
    logit(sp) = mu_lsp + id_lsp
    id_lse, id_lsp ~ biv.normal(0, sigma)
    Number of observations = 9
    Number of studies = 9

****************************************************************************************

****************************************************************************************

Between-study heterogeneity statistics
                           covar            rho
                            0.33             0.28
                           Tau.sq           I^2(%)
Generalized                 1.31            54.11
Sensitivity                 1.03            77.60
Specificity                 1.38            30.30

LR Test: RE vs FE model
         Chi2        degrees of
       statistic       freedom         p-val
        253.29          3             0.0000
****************************************************************************************
            Study specific test accuracy: Absolute Measures
****************************************************************************************
```

Study	Sensitivity Estimate	Sensitivity [95% Conf.	Interval]	Specificity Estimate	Specificity [95% Conf.	Interval]
2023 Krag, C	0.89	0.85	0.91	0.90	0.87	0.92
2023 Lee, K	0.73	0.67	0.79	0.91	0.87	0.93
2022 Bridge, C	0.96	0.91	0.99	0.87	0.79	0.92
2022 Tasci, B	0.99	0.95	1.00	0.99	0.98	1.00
2021 Liu, C	0.98	0.94	1.00	0.76	0.72	0.80
2021 Nael, K	0.90	0.86	0.93	0.97	0.95	0.98
2020 Duan, Y	0.76	0.56	0.90	1.00	0.03	1.00
2020 Federau, C	0.91	0.78	0.97	0.75	0.58	0.88
2019 Bizzo, B	0.96	0.85	0.99	0.97	0.95	0.99
Overall	0.93	0.86	0.96	0.93	0.84	0.97

Table S8b. STATA data from the metandi analysis on AI stroke detection in MRI.

```
metandi tp fp fn tn, detail

Refining starting values:

Iteration 0:  Log likelihood = -68.472586
Iteration 1:  Log likelihood = -67.509919
Iteration 2:  Log likelihood = -67.105527
Iteration 3:  Log likelihood = -67.083225

Performing gradient-based optimization:

Iteration 0:  Log likelihood = -67.083225
Iteration 1:  Log likelihood = -67.082997
Iteration 2:  Log likelihood = -67.082997

Mixed-effects logistic regression               Number of obs     =         18
Binomial variable: _metandi_n
Group variable: _metandi_i                      Number of groups  =          9

                                                Obs per group:
                                                              min =          2
                                                              avg =        2.0
                                                              max =          2

Integration points =   5                        Wald chi2(2)      =      66.07
Log likelihood = -67.082997                     Prob > chi2       =     0.0000

------------------------------------------------------------------------------
_metandi_t~e | Coefficient  Std. err.      z    P>|z|     [95% conf. interval]
-------------+----------------------------------------------------------------
 _metandi_d1 |   2.539721   .3752169     6.77   0.000     1.804309    3.275132
 _metandi_d0 |   2.530999   .4274954     5.92   0.000     1.693124    3.368875
------------------------------------------------------------------------------

------------------------------------------------------------------------------
  Random-effects parameters  |   Estimate   Std. err.     [95% conf. interval]
-----------------------------+------------------------------------------------
_metandi_i: Unstructured     |
               sd(_metan~1) |   1.012188   .3074672      .5580795    1.835804
               sd(_metan~0) |   1.173772   .3301926      .6762903    2.037203
     corr(_metan~1,_metan~0) |   .2763606   .4033303     -.5169318    .8142855
------------------------------------------------------------------------------
LR test vs. logistic model: chi2(3) = 253.28             Prob > chi2 = 0.0000

Note: LR test is conservative and provided only for reference.
```

Table S8b continued.

```
Meta-analysis of diagnostic accuracy

Log likelihood   = -67.082997                 Number of studies =        9
------------------------------------------------------------------------------
             |      Coef.   Std. Err.      z    P>|z|     [95% Conf. Interval]
-------------+----------------------------------------------------------------
Bivariate    |
  E(logitSe) |   2.539721   .3752169                      1.804309    3.275132
  E(logitSp) |   2.530999   .4274954                      1.693124    3.368875
Var(logitSe) |   1.024525   .6224293                      .3114527    3.370177
Var(logitSp) |   1.377741   .7751417                      .4573686    4.150198
Corr(logits) |   .2763606   .4033303                     -.5169318    .8142855
-------------+----------------------------------------------------------------
HSROC        |
      Lambda |   5.085277   .6276956                      3.855016    6.315538
       Theta |   .1922981   .5372345                     -.8606622    1.245258
        beta |   .1481082   .4013269     0.37   0.712     -.638478    .9346944
     s2alpha |   3.032832   1.829501                       .929772    9.892825
     s2theta |   .4298701   .2533246                      .1354316     1.36444
-------------+----------------------------------------------------------------
Summary pt.  |
          Se |   .9268799   .0254298                      .8586727    .9635658
          Sp |   .9262866   .0291893                      .8446345    .9667175
         DOR |    159.289   100.2884                      46.37341    547.1453
         LR+ |   12.57411   5.068631                      5.706336    27.70748
         LR- |   .0789389   .0281229                      .0392682    .1586872
       1/LR- |   12.66802   4.513131                      6.301706    25.46591
------------------------------------------------------------------------------
Covariance between estimates of E(logitSe) & E(logitSp)   .0364283
```
